# Affinity and competition for TBP are molecular determinants of gene expression noise

**DOI:** 10.1038/ncomms10417

**Published:** 2016-02-02

**Authors:** Charles N. J. Ravarani, Guilhem Chalancon, Michal Breker, Natalia Sanchez de Groot, M. Madan Babu

**Affiliations:** 1 MRC Laboratory of Molecular Biology, Francis Crick Avenue, Cambridge CB2 0QH, UK; 2 Department of Molecular Genetics, Weizmann Institute of Science, Rehovot 7610001, Israel

## Abstract

Cell-to-cell variation in gene expression levels (noise) generates phenotypic diversity and is an important phenomenon in evolution, development and disease. TATA-box binding protein (TBP) is an essential factor that is required at virtually every eukaryotic promoter to initiate transcription. While the presence of a TATA-box motif in the promoter has been strongly linked with noise, the molecular mechanism driving this relationship is less well understood. Through an integrated analysis of multiple large-scale data sets, computer simulation and experimental validation in yeast, we provide molecular insights into how noise arises as an emergent property of variable binding affinity of TBP for different promoter sequences, competition between interaction partners to bind the same surface on TBP (to either promote or disrupt transcription initiation) and variable residence times of TBP complexes at a promoter. These determinants may be fine-tuned under different conditions and during evolution to modulate eukaryotic gene expression noise.

## Abstract (web summary)

TATA boxes in gene promoters are associated with high level of cell-to-cell variation in gene expression. Through integration of multiple data sets, the authors now provide insights into how the interactions of TBP with DNA and other proteins can lead to noisy expression.

## Graphical abstract

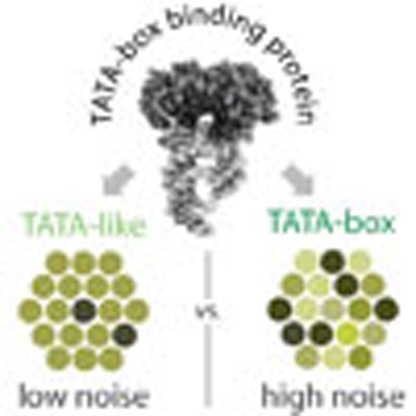

Gene expression noise is the measure of cell-to-cell variability in the expression level of a gene in a population of genetically identical cells that are grown in the same environment^1^. Differences in expression levels (noise) can result in phenotypic diversity between individuals despite genetic homogeneity^2^,^3^. Non-genetic variation as a basis for phenotypic diversity is an evolvable trait^4^ and is critical for development and disease^5^,^6^. Indeed, genome-wide studies have revealed that some genes are noisier than others^7^ and that stochastic variation in levels of regulatory proteins can generate phenotypic diversity^8^. In the last decade, an increasing number of factors that influence noise during transcription have been identified^2^,^9^,^10^. Specifically, variability in chromatin organization and transcription factor (TF) binding play a role by regulating access to the DNA by the transcription machinery^11^,^12^,^13^, thereby leading to differences in transcriptional output and noise (Fig. 1a). Although these factors provide an important mechanistic framework^14^, the molecular aspects of how the process of transcription initiation, the different assembly pathways of the transcriptional machinery, and their dynamics—the key steps on having access to the promoter—lead to noise remains less well understood.

When a promoter becomes accessible after chromatin reorganization and TF binding, RNA polymerase II can be recruited in different ways^15^,^16^,^17^. All assembly pathways require a conserved factor called the TATA-box binding protein (TBP), the scaffold for assembling the general transcription factors (GTFs) to form a pre-initiation complex (PIC)^18^,^19^. TBP recognizes a DNA element called the TATA-box^20^ (see Supplementary Fig. 1 for definitions of a TATA-box). Not all genes have a canonical TATA-box sequence, but TBP is recruited to all promoters to initiate transcription^21^. Crucially, genes with a TATA-box are associated with high noise^22^,^23^,^24^. In cells, TBP exists in different complexes (Fig. 1b) that either promote or disrupt PIC formation^25^; TBP can be a dimer, bound to the DNA, part of different co-activator complexes, or be engaged by PIC-disrupting factors such as Mot1p and NC2 (ref. 25). The competition between the interacting partners sequesters the ∼20,000 copies of TBP into distinct TBP-containing complexes^26^ that have distinct properties (for example, diffusion rates^27^ and residence times at the promoter^28^). Disrupting this dynamic equilibrium can influence the abundance of specific TBP complexes and affect gene expression globally^29^. Thus, how the TATA-box and the variability in the subsequent steps for PIC formation contribute to noise remains unclear. Here, we describe a molecular model of how TBP, the sequence of its binding site, the complexes it can form and their respective residence times at a promoter can make a gene more or less noisy through an integrated analysis of multiple large-scale data sets, computer simulation and experimental validation in yeast (Fig. 1c; Supplementary Methods).

## Results

### Functional TBP-binding sites are accessible at a promoter

**Figure 2:**
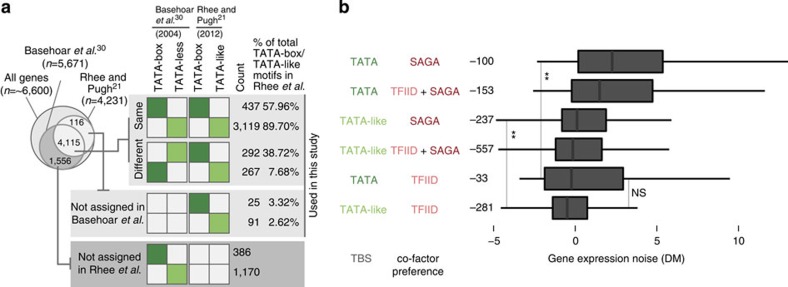
Comparison and classification of TBS sequences and the relationship between TBS type, co-activator binding preference and gene expression noise. (**a**) Comparison of promoter classification using the computationally inferred TATA-box containing promoters by Basehoar *et al.*^30^ and nucleotide-level resolution measurement of TBP-binding sites by Rhee and Pugh^21^. The Venn diagram on the top left indicates the number of genes analysed in the different data sets and the extent of overlap of the genes studied, respectively. The matrix on the right indicates the degree to which the more recent data set from Rhee and Pugh agrees (top) and disagrees (bottom) with the assignment of genes in Basehoar *et al.*^30^ to host a TATA-box (dark green) or a TATA-like (light green) sequence/TBP-binding site (TBS) in their respective promoters. Genes with no assignment in both studies are also shown. (**b**) The relationship between promoter TBS type and co-activator preference, and gene expression noise. The respective distributions in terms of gene expression noise (DM) are shown as box plots and were ranked according to the median noise value of each class. In the different panels, statistical significances between distributions of medians were assessed using the Wilcoxon rank-sum tests that were corrected for multiple testing (‘**' for *P*<0.01 and ‘NS' for not significant). The number of genes in each class is given on the left of the plot.

Previously, genes were classified as TATA-box containing or TATA-less based on predicted TATA-box sequences somewhere in their promoter^30^. Recently, TBP was found to bind at the promoter of nearly every gene, and the exact location of functional TBP-binding sites (TBS) was determined at nucleotide resolution^21^. This allowed a new classification of genes into those containing a TATA-box and those with an experimentally verified TATA-like sequence that are very similar to a TATA-box with no more than two mismatches^21^ (Supplementary Fig. 1; Fig. 2a). Using the new scheme and the increased resolution in defining a functional TBS (that is, where a PIC forms), we observed that the TBP-binding site of ∼80% of TATA-box genes and ∼90% of the TATA-like genes are not occluded by a well-positioned nucleosome. This means that a TBS is more likely to be accessible to the transcriptional machinery in both promoter types (Supplementary Fig. 1; Supplementary Note 1), raising the question as to how an accessible TATA-box and TATA-like TBS influences PIC formation and noise.

### TBS type is linked with cofactor binding preference

At an accessible promoter, TBP forms transcriptionally permissive assemblies either as part of a TFIID or SAGA co-activator complex. TFIID is a ∼1.2 MDa complex^31^ of which TBP has been observed to be a constitutive subunit^19^. SAGA is also a multi-subunit complex^32^,^33^ (∼1.8 MDa), but unlike TFIID, TBP only weakly binds to SAGA, and is less likely to be a constitutive subunit^16^,^34^. In contrast to TFIID, TBP and SAGA subunits can arrive at a promoter independently, where they interact relatively weakly^16^,^32^,^35^. Thus both co-activator complexes lead to PIC formation but in different ways. While some sequence specific TFs and nucleosome modifications influence the differential recruitment of the co-activator complexes^35^,^36^, the TBS may play a role in their ability to assemble at a promoter. Using the new classification scheme, we observed that there is a continuum of binding preference for the co-activator complexes: genes with a TATA-box show predominant binding by SAGA and genes with a TATA-like sequence show predominant binding by TFIID. Interestingly, both co-activators bind to the same promoter of several genes, suggesting the existence of two sub-populations^37^ where either TFIID or SAGA is bound in different individuals in the promoter of a given gene (see Supplementary Fig. 2 and Supplementary Methods for how the co-activator-regulation classes were defined; note that the percentage of gene classes do not represent the true biological proportions but reflects the fact that they were classified according to the median occupancy value).

### Cofactor binding preference is linked to noise

In terms of noise (measured as coefficient of variation (CV); ratio of s.d. to mean abundance), we observed that TATA-box genes are noisier and display higher expression level than the newly classified TATA-like genes (Supplementary Fig. 2). In fact, TATA-box genes that are highly expressed display a higher CV, suggesting that the higher noise cannot simply be explained due to low protein abundance (mean-CV inverse relationship^38^). Since the mean abundance tends to be inversely related to CV^38^, we subsequently used DM, which is an abundance-independent measure of cell-to-cell variability (DM; distance from median CV; herein referred to as noise - except when specified otherwise; Supplementary Fig. 2). We found that TATA-box genes bound by SAGA have higher noise^7^ (Fig. 2b). Irrespective of the TBS type, TFIID bound genes show comparably low noise. Among the genes with a TATA-like TBS, those bound by SAGA display higher noise compared with the ones bound by TFIID. This suggests that the specific co-activator that assembles at a promoter influences noise despite the type of TBS. Collectively, these observations suggest that the TBS type is linked with preferential co-activator assembly, and the specific co-activator that assembles at a promoter may influence noise (Fig. 2b). This raises the following questions: what is the molecular explanation for the different TBS types to preferentially assemble SAGA or TFIID? And why do SAGA-regulated genes tend to be noisier and TFIID-regulated genes tend to be less noisy?

### TBP displays distinct preference for TBS sequences

**Figure 3:**
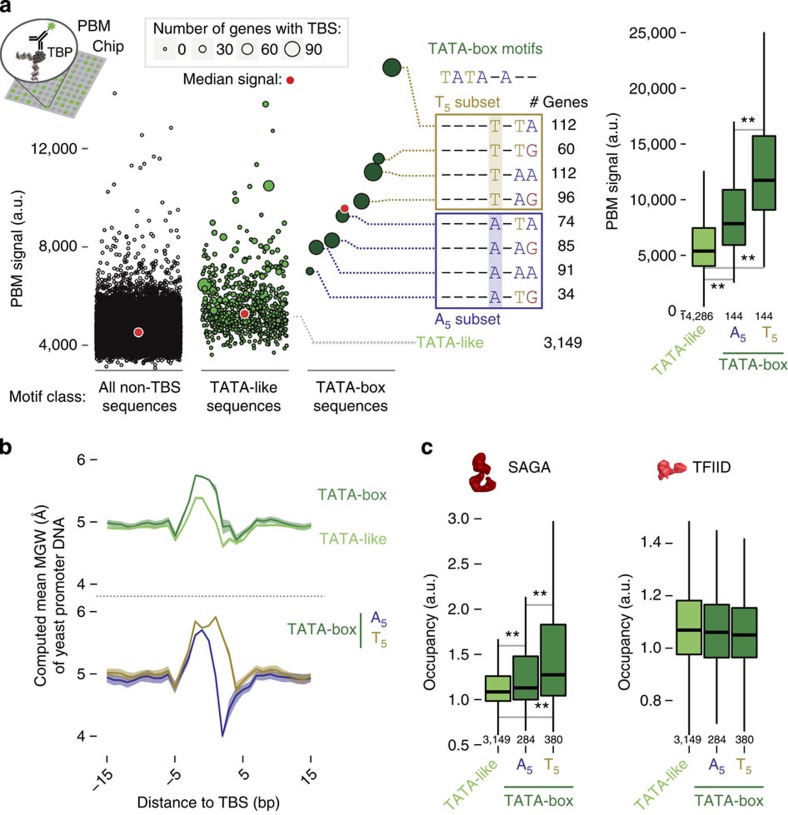
TBP binding affinity and intrinsic DNA flexibility of TBS sequences. (**a**) The protein binding microarray (PBM) signal intensity is plotted for the different TBS type sequences (dark green for TATA-boxes and light green for TATA-like sequences) and all other 8-mer sequences (black). Every circle represents the median signal measured for all the repeats on the chip for that given sequence, and the size indicates the number of promoters in yeast that host this particular sequence in its TBS. The red dot indicates the median PBM signal per sequence class. PBM signals for TATA-box subtypes (middle), that is, T_5_ (yellow) and A_5_ (blue). The number of genes with the given sequence in the TBS in yeast is shown on the right. Box plots of distributions of PBM signal intensity for probes hosting the different TBS motifs (right). (**b**) Computed intrinsic minor groove width (MGW) for TATA-box (dark green) and TATA-like sequences (light green; top panel). The shaded area represents confidence intervals. MGW for T_5_ (yellow) and A_5_ (blue) subtypes are shown in the bottom. (**c**) Genes were classified based on the TBS type in their promoter (dark green for TATA-box with the T_5_ and the A_5_ subsets, light green for TATA-like sequences) and box plots of distributions for SAGA and TFIID occupancies are shown. In all the panels, Wilcoxon rank-sum tests (that were corrected for multiple testing; ‘**' for *P*<0.01) were performed to assess statistical significance between the medians of the distributions. The number of genes in each class is given on the bottom of the box plot.

Since TBP is recruited differently at genes that are regulated by the two co-activators, we investigated how the chemical differences and intrinsic flexibility of the TBS sequences (properties that affect affinity and kinetics^39^,^40^,^41^) might influence co-activator binding. An analysis of protein-binding microarray (PBM) data containing all possible 8-mer sequences^42^,^43^ showed that the signal intensity for monomeric TBP was higher for TATA-box containing probes (compared with TATA-like or other 8-mers; Fig. 3a; left). Intriguingly, we observed a large spread of PBM intensity among the distinct TATA-box sequences. Upon ordering them by their PBM intensity, the sequences naturally cluster into two subsets: those with a Thymine or Adenine in position 5 (T_5_/A_5_ subset) of the TATA-box (Fig. 3a; middle). The PBM intensity is higher for the T_5_ compared with the A_5_ subset (Fig. 3a; right). An analysis of published binding kinetics data^44^ showed that there is no statistically significant difference in the ‘on' and ‘off' rates for TBP binding to the different TBS sequences (Supplementary Fig. 3).

### TBS flexibility may determine TBP binding preference

An analysis of the available structures of TBP in complex with different TBS sequences revealed that (a) the TBP structure in the DNA bound and the unbound forms are highly similar (irrespective of either a TATA-box or TATA-like sequence), (b) the DNA of both TBS types are bent to the same extent and (c) the complexes are very similar in terms of buried surface area, interaction energy and number of interacting residues (Supplementary Fig. 3). Despite this high degree of similarity in the DNA structure of the bound complexes, the computed minor groove width (MGW; an indicator of DNA conformational flexibility^45^; Supplementary Note 2) of the yeast promoter sequences showed that TATA-box sequences are likely to adopt a wider MGW (Fig. 3b)^46^. This indicates that TATA-box sequences are more flexible than TATA-like sequences and hence energetically easier to bend to form the final configuration (Fig. 3b). Furthermore, the relatively wider MGW extends throughout the whole motif for the T_5_ subset, whereas, there is a sharp drop for the A_5_ subset, possibly explaining the higher *in vitro* binding of monomeric TBP in the T_5_ subset (Fig. 3b). These observations collectively suggest that the bendability of the TBS (rather than the chemical differences) might play a role in the thermodynamics of monomeric TBP binding preference for different TBS sequences.

### TBP binding affinity drives co-factor assembly

The preferential binding of TBP to the different TBS sequences can influence the assembly of co-activator complexes. Because TBP can arrive independently at SAGA-regulated genes^35^, such promoters may harbour high-affinity TBS sequences compared with TFIID-regulated genes. Consistent with this, SAGA occupancy *in vivo* was higher in promoters of the T_5_ compared to the A_5_ subset (Fig. 3c). In contrast, TFIID occupancy remained comparable across promoter types, including TATA-like sequences (Fig. 3c). This suggests that *in vivo*, TBP as a part of TFIID can bind both TATA-box and TATA-like promoters equally well despite the intrinsic preference for monomeric TBP to bind TATA-box sequences. This may be because the other subunits of TFIID make additional contacts and stabilize the TBP:TBS complex once recruited^47^,^48^. These observations highlight that SAGA assembly is linked to the differential affinity of monomeric TBP to bind specific TBS sequences, and suggests a role for TBS bendability in the preferential assembly of SAGA at TATA-box promoters *in vivo*.

### TBP in TFIID is inaccessible by Mot1p

**Figure 4:**
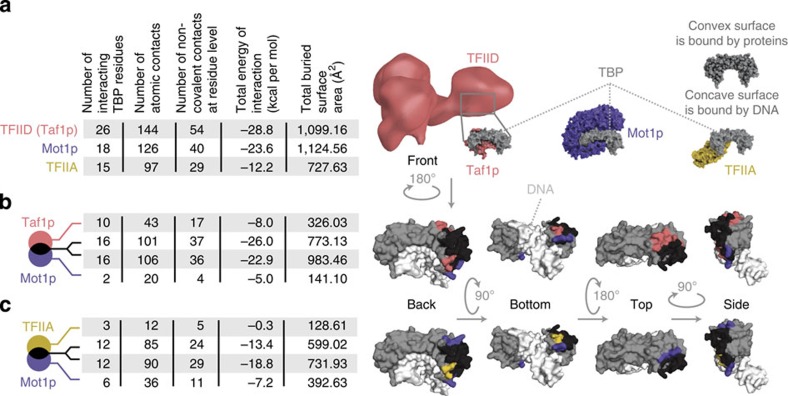
Properties of the interface and interactions between TBP and its partner proteins. (**a**) Characterization of the interaction interface of TBP in complex with distinct PIC-influencing factors. TBP (grey) is shown from the same orientation as it interacts with TFIID (pink), Mot1p (purple) and TFIIA (yellow). For TFIID, the electron microscopy structure is shown. One of the subunits (Taf1p) with two TAND domains that interact with TBP is also highlighted. The properties calculated to characterize the interaction interface include the number of interacting TBP residues, the number of atomic contacts, the number of non-covalent contacts, the total computed energy of interaction (kcal per mol), and the total buried surface area (Å^2^). (**b**) Comparison of the TFIID(Taf1p):TBP interface and the Mot1p:TBP interface (convex TBP surface only). The contributions of the different factors are divided into those that are unique to each (pink for TFIID and purple for Mot1p) and those that are common/overlapping (black). (**c**) Comparison of the TFIIA:TBP interface and the Mot1p:TBP interface (convex TBP surface only). The contributions of the different factors are divided into those that are unique to each factor (yellow for TFIIA, purple for Mot1p) and those that are common/overlapping (black). In both (**b**,**c**) the interacting regions are mapped onto the structure and viewed from different angles. The DNA is coloured white.

Having investigated the role of the TBS sequences, we then studied how the TBP interaction partners influence PIC formation. We analysed the structures of TBP in complex with its different binding partners and characterized the nature of the interaction surface (Fig. 4a) after establishing the equivalent TBP residues between the different structures (Supplementary Fig. 4). A key factor that can disrupt PIC formation is Mot1p, which binds and evicts monomeric TBP from the DNA^49^. Mot1p only requires access to the convex surface of TBP^50^. However, low-resolution EM models of TFIID show that TBP is buried within the complex (Fig. 4a)^47^,^51^ and cannot be readily accessed by Mot1p. A comparison of the structure of TBP in complex with the TAND domain of Taf1p (TBP interacting subunit of TFIID) revealed that Taf1p-TAND and Mot1p overlap significantly in terms of the interaction surface on TBP (16 overlapping residues; ∼800 Å^2^). Furthermore, Taf1p makes more unique contacts with TBP than Mot1p (Fig. 4b). Therefore, when TBP is recruited as a part of TFIID with its subunits, Mot1p is unlikely to readily displace the subunits of TFIID and gain direct access to TBP in the complex, especially, given the nanomolar affinity for TBP to bind certain subunits of TFIID^34^. Thus, at TFIID-regulated promoters (more likely to harbour a TATA-like TBS; Supplementary Fig. 2), Mot1p is unlikely to directly interfere with PIC formation.

### Mot1p can compete with SAGA for TBP

When monomeric TBP binds the TBS, TFIIA is required to stabilize the TBP:DNA complex^52^,^53^ for SAGA assembly. Mot1p and TFIIA both bind to the convex surface of monomeric TBP^50^,^54^, although Mot1p binds more extensively than TFIIA (Fig. 4a). Despite the overlap in binding, Mot1p uniquely contacts twice as many residues as TFIIA, suggesting a stronger interaction between Mot1p and TBP (Fig. 4c). Although additional regions of TFIIA can contact TBP, they are likely to be transient as these regions have missing electron density^55^. The transient interactions are however important for PIC formation irrespective of the co-activator recruited^47^,^55^. Thus, at SAGA-regulated promoters (more likely to harbour a TATA-box TBS; Supplementary Fig. 2), Mot1p can compete with TFIIA and hence, SAGA assembly. This can disrupt PIC formation, and thereby prevent transcription initiation.

### Mot1p binding at promoters with distinct TBS types

**Figure 5:**
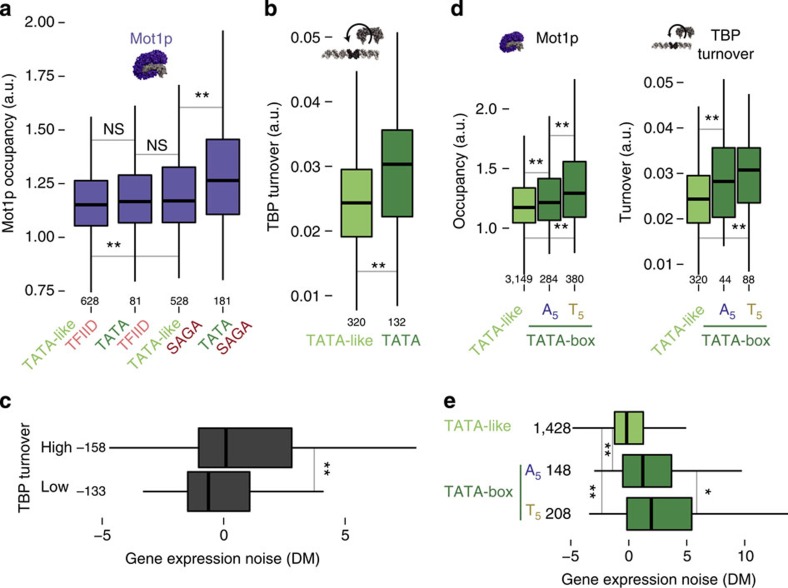
Box plots of the distributions highlighting the relationship between TBS, TBP interacting proteins, TBP turnover and gene expression noise. (**a**) Mot1p occupancy at promoters with different TBS types and different co-activator regulation classes. (**b**) TBP turnover at promoters with different TBS types (dark green for TATA-box and light green for TATA-like). (**c**) The TBP turnover in promoters of genes was split into low and high bins ([0.008–0.026] and (0.026–0.051] a.u., respectively) based on the median TBP turnover and the distributions of gene expression noise for these two classes are displayed as box plots. Genes were classified based on the TBS type in their promoter (dark green for TATA-box with the T_5_ and the A_5_ subsets, light green for TATA-like sequences) and the distributions of Mot1p occupancy and TBP turnover are shown in (**d**) and noise in (**e**). In all the panels, Wilcoxon rank-sum tests (that were corrected for multiple testing; ‘**' for *P*<0.01, ‘*' for *P*<0.05) were performed to assess the statistical significance of the differences between the medians of the distributions. The number of genes in each class is given on the bottom or the left of the box plot.

An analysis of genome-wide binding data revealed that Mot1p occupancy is higher at TATA-box promoters where monomeric TBP can bind. Mot1p occupancy is also higher at SAGA occupied promoters (Fig. 5a; Supplementary Fig. 5). This suggests that Mot1p and SAGA occupy the same promoter. However, the structural data above suggests that Mot1p and TFIIA (required for SAGA assembly) cannot bind to the same TBP molecule as they compete for the same surface on TBP, which has also been inferred by an earlier study^56^. One explanation that is consistent with both observations is the existence of two sub-populations of cells; one harbouring the transcriptionally non-permissive Mot1p:TBP:TBS complex (which will be transient and readily dissociate) and the other harbouring the permissive SAGA:TBP:TBS at the same promoter. This heterogeneity in distinct TBP assemblies between different individuals may lead to variable transcriptional output. Intriguingly, we note that genes that are regulated by both TFIID and SAGA have the highest Mot1p occupancy. These genes tend to be highly expressed, highly regulated by a larger number of distinct TFs, and have a highly dynamic chromatin at the promoter (Supplementary Fig. 5). This suggests that the promoter DNA might be more often accessible and might expose non-functional TBP-binding sites, which may lead to spurious and inappropriate TBP binding that needs to be cleared up by Mot1p (Supplementary Fig. 5). By evicting TBP from such non-functional sites, Mot1p can facilitate binding of TBP to the appropriate sites, and may promote transcription at some promoters^29^,^57^. In line with this, we observe that the promoters of these genes have higher TBP binding in regions that are distal to the PIC forming TBS and might explain the unexpectedly high Mot1p occupancy in the region (Supplementary Note 3). Finally, consistent with the observations that Mot1p may not have access to the TBP within TFIID, we found that Mot1p occupancy is lowest at TFIID-regulated promoters, irrespective of the TBS type (Fig. 5a). Thus, when TFIID is assembled at an accessible TBS, PIC formation is unlikely to be disrupted by Mot1p. Therefore, individual cells in a population might show little variability in PIC formation, thereby leading to a consistent transcriptional output.

### TBP residence times are longer at TATA-like promoters

The extent of switching between the different TBP complexes at a promoter will depend on the residence time of the respective complexes. Investigation of TBP turnover data using the new TBS classification scheme revealed that the turnover rate is lower in TATA-like promoters^28^ (Fig. 5b). This suggests that the same TBP complex is present for a longer period of time. This is consistent with the structural and genome-scale occupancy data that TATA-like promoters are more likely to be bound by TFIID that might prevent TBP removal by Mot1p. In line with this, genes with low TBP turnover display low noise (Fig. 5c). Thus, for TATA-like promoters, it seems that lower noise might be a consequence of the stable association of TBP as part of TFIID, leading to a consistent transcriptional output. At TATA-box promoters, TBP turnover is higher, which suggests that switching between TBP complexes happens frequently (Fig. 5b). This raises the question as to how high TBP turnover can lead to high noise.

### Rapid TBP recycling by Mot1p leads to higher noise

To investigate why high TBP turnover leads to high noise, we analysed Mot1p occupancy in the different TATA-box subtypes. Mot1p occupancy was higher for the T_5_ compared to the A_5_ subset (Fig. 5d), which in turn is linked to the increased binding of TBP (Fig. 3a). Thus, at TATA-box promoters, monomeric TBP readily binds the high-affinity TBS and Mot1p might rapidly evict it (Fig. 5d). This leads to a futile cycle where TBP is rapidly recycled and the promoter is transcriptionally silent until SAGA binds. Indeed, depletion of Mot1p leads to increased TBP binding preferentially at TATA-box promoter^29^. The variability in the time spent in the transcriptionally silent state and the competition to switch to the permissive state on SAGA binding, can lead to variability in the timing of transcription initiation and therefore, cell-to-cell differences in gene expression levels. Collectively, these observations highlight that at an accessible promoter, the affinity of TBP to the TBS sequence provides the context for noise to emerge. Consistent with this, we find that the binding preference of monomeric TBP for the different TBS types is linked with the extent of noise (Fig. 5e).

### Simulations to explore possible noise regimes

**Figure 6:**
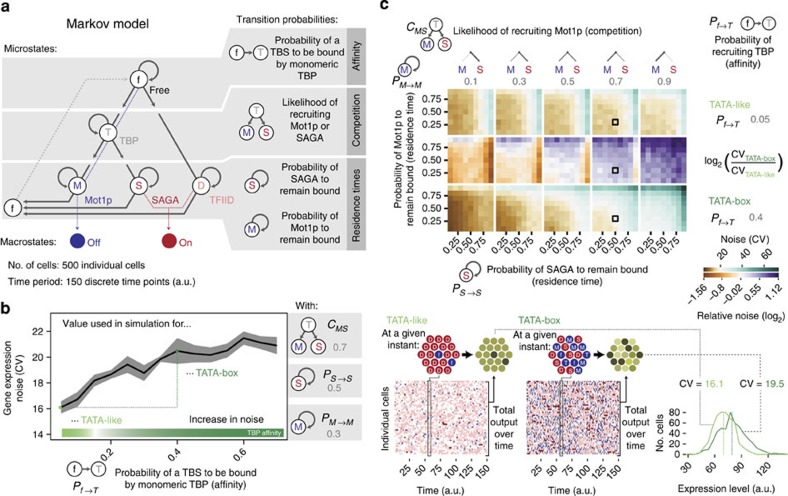
Stochastic simulations highlighting how TBP affinity for a TBS, competition between Mot1p and SAGA and their residence times influence noise. (**a**) The possible TBP assemblies at the promoter (microstates) leading to transcriptional output (On state) or no output (Off state) and the transitions between the microstates were used to build a Markov model. The simulation was performed for a cell population with 500 individual cells and for 150 time points. (**b**) The intrinsic binding affinity of TBP for different TBS sequences and its relationship with noise. For this simulation, the competition and residence time parameters for Mot1p and SAGA were kept constant. The grey ribbon around the trend line indicates plus and minus one s.d. from the mean from three independent simulations. (**c**) Phase diagram of the possible noise behaviour for different parameters for a promoter under different co-activator and Mot1p regulation. Black square boxes in the matrix highlight the parameter combination that was used to generate the simulation results shown in **b**. In the bottom, the history of microstates from the simulation for a TATA-box and TATA-like promoters is shown (left). At a given instant, individual cells with TATA-like promoter have a more homogenous distribution of microstates and a corresponding consistent expression output. In contrast, cells with TATA-box promoters show a more heterogeneous distribution of microstates and a corresponding variable expression output (right). The respective mean expression values are indicated with a dashed line in the distribution.

Integrating the observations from the biochemical, biophysical, structural and genome-scale occupancy data, we performed a discrete-time stochastic simulation of transcription initiation from an accessible TBS to explore the role of (a) differential affinity of TBP to TBS sequences, (b) competition between TBP interaction partners (specifically, the extent of Mot1p/SAGA assembly) and (c) variable residence time of the TBP complexes (Mot1p and SAGA residence time in particular) on noise (Fig. 6a). The simulation assumes the promoter context to be the same (that is, TF binding and nucleosome organization are not modelled explicitly). This provides an opportunity to monitor how the different TBS sequences alone could affect the different TBP complexes (microstates) that can assemble at a promoter in individual cells, which in turn impacts the transcriptional output (On/Off macrostates), thereby influencing noise in a cell population (Fig. 6a; Supplementary Methods).

First, we modelled the effect of the affinity of TBP to different TBS sequences (affinity parameter) in situations that are reflective of the observations described above, that is, (a) Mot1p is more likely to outcompete SAGA (competition parameter), (b) longer and intermediate residence times for TFIID and SAGA, respectively (residence time parameter) and (c) lower residence time for Mot1p at promoters since Mot1p enzymatic activity is high in cells (Mot1p:TBP:DNA complex dissociates rapidly; see also Supplementary Note 4 and Fig. 6b). The simulations revealed that noise increases as the probability to assemble monomeric TBP increases. A more comprehensive simulation that systematically varied the competition and residence times of Mot1p and SAGA revealed the landscape of noise values that are attainable (Fig. 6c). The simulations revealed that the waiting time between the transcriptional On states is longer for TATA-box genes in situations when Mot1p outcompetes SAGA. The longer waiting time is a consequence of rapid recycling of monomeric TBP between the unbound form and the TBP:TBS complex (Off state) due to Mot1p, resulting in higher TBP turnover (Supplementary Fig. 6). The variability (between different individuals) in switching to the occasional On state with large expression burst via SAGA leads to differences in the expression output, resulting in higher noise.

Importantly, the simulations also revealed the existence of situations where TATA-box genes have lower noise than TATA-like genes. For instance, when SAGA outcompetes Mot1p (for example, under low abundance of Mot1p and/or high abundance of SAGA), a TATA-box gene is less noisy since SAGA more often wins the competition and/or SAGA residence times are higher. This can happen when a strong transcriptional activator effectively recruits and tethers SAGA to the promoter (for example, SAGA recruitment by Gal4p^36^). In such situations, the influence of Mot1p is minimized and the promoter is more often in the On state in TATA-box genes, thereby resulting in a stable transcriptional output, resulting in low noise.

### Experimental validation

**Figure 7:**
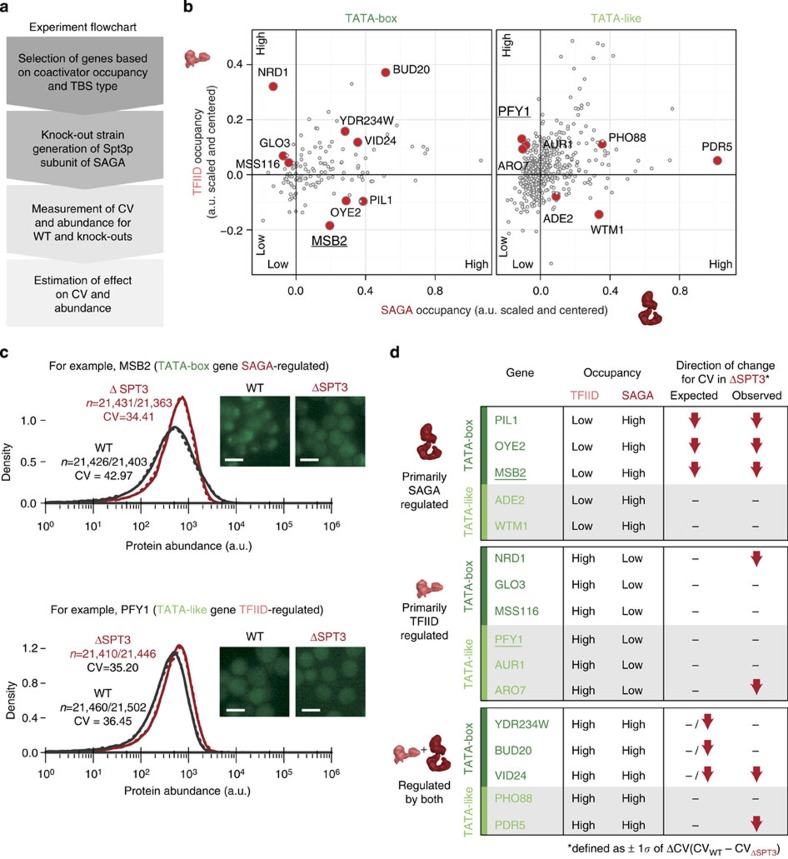
Deletion of SAGA impacts noise in the way predicted by the model. (**a**) The experimental pipeline used in this study. (**b**) The genes were selected based on SAGA and TFIID occupancy for both TBS types (TATA-box containing, dark green and TATA-like sequence, light green). The selected genes can be classified into six groups with at least two genes in each group (total of 16 genes; red dots). (**c**) Fluorescence microscopy images of two genes (MSB2: TATA-box TBS and predominantly regulated by SAGA; PFY1: TATA-like TBS and predominantly regulated by TFIID), in the wild type (WT) strain and SAGA knockout strain (Δ*SPT3*; *SPT3* of the SAGA complex interacts with TBP). Scale bars, 4.2μm. Flow cytometry measurements of thousands of single cells (∼20,000 cells; no. of cells, *n* are shown for both replicate experiments) were recorded with two replicates to get the distribution of expression levels in the population and to investigate the impact of the Δ*SPT3* knockout on noise (CV). (**d**) Expected and observed effect of *SPT3* knockout (SAGA subunit) for genes with different core promoter types. Genes are classified based on their TBS type (TATA-box, dark green and TATA-like, light green), and their respective TFIID and SAGA occupancy (high/low). The expected effects of the Δ*SPT3* knockout based on the mechanistic model (no change; dash or a decrease in noise; red down arrow) are shown. The observed decrease in effect was defined as a deviation from WT noise levels by at least one s.d. from all measured differences.

We carried out experiments in yeast (Fig. 7a) to test whether lowering the abundance of SAGA impacts noise in a way predicted by the model (see Discussion). For this, we measured noise (as measured by CV; Supplementary Note 5) for 16 different yeast genes in two different genetic backgrounds (wild-type and SAGA/Spt3p mutant; Fig. 7b and Supplementary Fig. 7). We selected the Spt3p subunit of SAGA as it is a non-essential subunit that binds TBP and hence likely to compete with Mot1p^32^. The individual genes were chosen to cover all the different combinations of core promoter properties in terms of the TBS type and TFIID/SAGA occupancy (Fig. 7b). The experimental findings are in agreement with what is expected from our model for the SAGA-regulated genes; lowering the level of SAGA does influence noise in the way as the model would predict, that is, a larger reduction of noise is observed in TATA-box genes compared with TATA-like genes on deletion of SAGA (Fig. 7c,d; Supplementary Fig. 7). For a majority of the genes belonging to the TFIID/SAGA+TFIID co-regulator classes, the findings are consistent. One reason why a small subset of genes does not show the expected behaviour might be due to gene specific mechanisms (that is, the extent of chromatin regulation, nucleosome context and TF binding) that may influence any of the steps in the model and hence can modulate noise (Supplementary Note 5). Finally, although we did not measure the variation in the abundance of the SAGA complex between individual cells from the wild-type population, it is reasonable to infer from our experiments that variation in the abundance of the SAGA complex (extrinsic noise) will affect the expression variability of SAGA-regulated genes.

## Discussion

By integrating measurements made at the whole population level with data on single-cell measurements, we provide insights into how TBP, the sequence of its binding site, the complexes it can form and their respective residence times at a promoter can make a gene more or less noisy. The findings fit within the existing framework of nucleosome organization and TF recruitment, and provide molecular insights into how the TBS can play an important role in influencing noise by determining the assembly pathway of transcriptional initiation.

**Figure 8:**
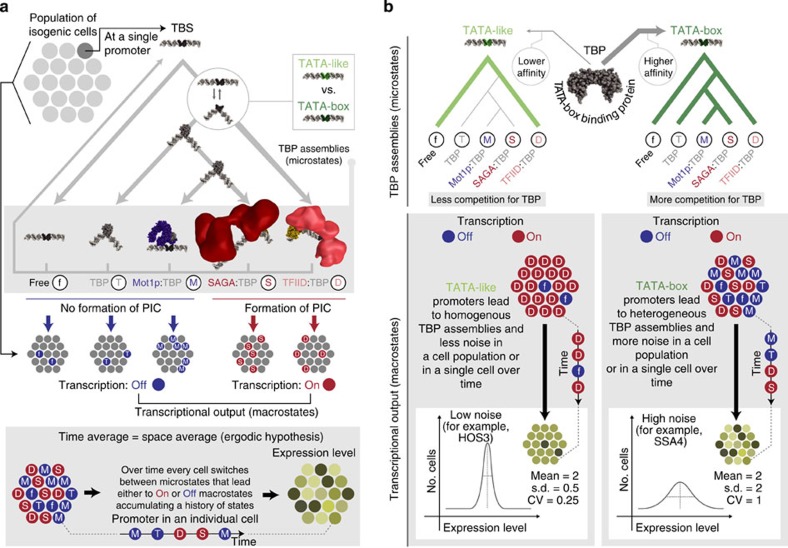
Molecular and mechanistic model for how the TBS in a promoter can lead to different TBP assemblies in a population of cells and thereby lead to noise in gene expression levels. (**a**) The TBS within a gene's promoter in a cell population can harbour different TBP assemblies following a given path through a chain of events/decision tree (top). A promoter can harbour a number of possible microstates: free (f), TFIID (D), TBP (T), SAGA (S) or Mot1p (M). Depending on the assembled TBP complex, the promoter will either be transcriptionally active or silent. The extent of switching between transcriptionally permissive or non-permissive states either (i) over time in an individual (time-average) or (ii) at an instance between individual cells in a population (space-average) determines the extent of expression noise (bottom). (**b**) TBS type determines the TBP complexes that can be assembled and thus can explain the emergence of noise. A TATA-box TBS can host a larger variety of microstates with different transcriptional output (top-right), which leads to more variability in gene expression levels between individual cells in a population. TATA-like TBS predominantly end up in the TFIID microstate, which leads to consistent expression output (top-left) and less variability in expression levels between individual cells in a population. In a population of cells, the different individuals might switch between the different TBP assemblies at a promoter and this will manifest as differences in the expression level of a gene (dark yellow for low abundance and light yellow for high abundance).

Our observations can be synthesized into the following model (Fig. 8) and can help rationalize a number of previously published perturbation studies (Supplementary Discussion). Depending on the bendability of certain DNA sequences, the affinity of TBP to bind a sequence might increase due to lowering the conformational strain to form the bound complex. Based on the affinity for certain TBS sequences, TBP may bind as a monomer or as a part of TFIID. If TFIID is recruited, the PIC is formed and transcription is initiated (On macrostate). If monomeric TBP binds, Mot1p or other TBP remodelling factors (for example, NC2) might evict TBP, resulting in no transcriptional output (Off macrostate). Alternatively, the SAGA complex might assemble and initiate transcription (On macrostate). Thus, at a TBS, TBP can exist in at least four microstates: (a) TFIID:TBP:TBS, (b) SAGA:TBP:TBS, (c) Mot1p:TBP:TBS or (d) TBP:TBS complex (Fig. 8a). Some microstates will have a higher residence time (for example, TFIID:TBP), whereas others are turned over quickly (for example, Mot1p:TBP), thereby influencing the transcriptional output^15^,^58^. Hence at a given time point, the same promoter could sample different microstates in different individuals or a single individual could sample different microstates over a period of time (ergodic hypothesis; that is, the behaviour averaged over time is the same when averaged over the space of all states; Fig. 8a, bottom)^59^.

At TATA-like TBS, TBP can bind only as a part of TFIID as the affinity of monomeric TBP is lower. Since TBP within TFIID is not accessible by Mot1p and the additional TFIID subunits contact the promoter extensively^48^, it remains bound for longer periods of time, thereby ensuring consistent and stable transcriptional output^60^,^61^,^62^. Thus, in a cell population, TATA-like TBS are likely to be stably occupied by TFIID and display less variability in expression levels between individuals (Fig. 8b). At TATA-box promoters, the affinity for monomeric TBP is higher and distinct TBP complexes can be assembled. In some individuals, TBP can bind as a monomer or as part of TFIID (in which case the outcome is similar to above). When TBP binds as a monomer, competition between Mot1p, GTFs and SAGA leads to the assembly of mutually exclusive TBP complexes at a promoter. Since TBP has a higher affinity for TATA-box TBS and Mot1p readily evicts monomeric TBP, this leads to frequent cycling between transcriptionally silent states (futile cycle), making the promoter responsive when SAGA is recruited. The variation in the waiting time to switch to the On state via SAGA between individuals will determine the extent of noise in a cell population (Fig. 8b). The history of the micro- and macrostates sampled at a promoter over time by an individual will determine the total abundance, burst size, burst frequency and the extent of variability in the expression level of a gene between individuals in a population (Supplementary Fig. 6).

Although the observations are consistent with a number of genes, the reported trends are unlikely to apply to every single gene. Several factors can influence (or override) the TBP complexes that can assemble at a promoter (Supplementary Discussion). However, since the mechanisms and components involved in transcriptional initiation are evolutionarily conserved, the reported observations are likely to be general and hold for other eukaryotes, including humans. Nevertheless, there will be differences in the number and types of distinct TBP complexes that can be formed at a promoter^17^. For instance, there are several paralogs of TBP in humans^63^, homologous protein complexes of SAGA (for example, ATAC), and splice isoforms of the PIC components in other eukaryotes^64^. This may result in an increase in the number of different TBP complexes (and the extent of switching between the different assemblies) in distinct cell types or during development, thereby providing an opportunity to tune the expression noise of individual genes or a subset of genes. An important implication of our findings is that in addition to alterations in the expression level of TBP or mutations in the TBS, variation in the expression level of TBP-interacting proteins (for example, SAGA, Mot1p and NC2) will globally influence noise by affecting the abundances of distinct TBP complexes and can thus be considered as global regulators of noise. Finally, the principles described here may represent a more general framework that is applicable to every major step along the process of gene expression. The interplay between affinity, competition for an essential regulatory factor and their residence times can drive the assembly of distinct complexes in different individuals of a cell population. This may lead to heterogeneities in the assembly of gene expression machineries, resulting in expression variability in a cell population.

## Methods

### Genome-wide data set on gene expression noise

Gene expression noise data were obtained from Newman *et al.*^7^ Noise values for every gene were computed as the ratio of the s.d. over the mean of fluorescence intensity for the entire cell population (CV) and the data was normalized to arrive at an abundance-independent measure of noise, called distance from the running median CV (DM). We used 1,804 protein-coding genes for which other genome-wide information was available, and for all calculations we used the DM values in yeast peptone dextrose (YPD) conditions as noise value.

### TBS classification

The TBP-binding site (TBS) classification status (TATA-box or TATA-like) for 4,231 mRNA-coding genes was obtained from Rhee and Pugh^21^. The main improvement of this data set is the base-pair resolution of localization of TBP on a genome-wide scale. This data set classified genes into either TATA-box genes if the motif was ‘pure', or TATA-like genes if their promoter hosts a TATA-box motif with up to two mismatches.

### Occupancy of TBP interaction partners in promoter regions

The genome-wide binding profiles of TBP, Mot1p, TFIID (Taf1p subunit), SAGA (Spt20p subunit) and Pol II across the entire yeast genome were acquired from van Werven *et al.*^65^ who employed chromatin immunoprecipitation (ChIP)—chip using cells in their exponential growth phase. The genomic probe enrichment at time point 0 was used and for each gene. The median factor occupancy was computed for probes situated in the promoter region.

### Gene classification based on co-activators in promoters

Every gene was classified according to whether it was TFIID regulated or SAGA regulated. As an intuitive measure of regulation, genes that have an occupancy value above the median of all genes' promoters for a factor (Taf1p or Spt20p) are considered to be regulated by that factor. Genes with an occupancy value below the median are considered as not regulated by that factor. This classification for TFIID regulation and SAGA regulation, respectively, leads to four possible states for a promoter: TFIID and SAGA regulated (+/+), only TFIID regulated (+/−), only SAGA regulated (−/+) and neither TFIID nor SAGA regulated (−/−). Finally rescaling and centring was applied for visual clarity and does not have an effect on calculating the respective median of SAGA and TFIID occupancy. Genes where either factor could not be detected were excluded from the analysis.

### Intrinsic TBP binding preference from PBM experiments

The raw data of the PBM chips was obtained from the Bulyk group website (http://the_brain.bwh.harvard.edu/uniprobe/downloads.php). The authors generated a microarray chip with a set of synthetic double-stranded DNA sequences that together represent all possible 10-mers of DNA. This unbiased set of sequences on the PBM chip can then be assessed for their protein binding levels, when incubating the chip with a GST-tagged protein. Binding of TBP to the DNA probes was detected using a GFP-tagged antibody to the GST, which in turn indicates the intrinsic binding preference of the TBP for all possible 8-mer sequences (the sequence length that is bound by TBP). To deconvolute and approximate which of the contained motifs is bound by TBP (every probe host multiple motifs), the median value of the many replicate measurements of the same motif on different probes was calculated. This strategy has been shown to be a good indicator^42^,^43^ of the signal and is informative of TBP's intrinsic binding preference to specific sequences. The motifs in the probes were then classified based on their TBS sequence types (TATA-box, TATA-like sequence and other 8-mers).

### Promoter DNA shape

The intrinsic MGW of all TBS sequences in the promoter context was calculated with DNAShape^46^. The method computes structural properties of DNA segments including MGW, which highlights the distance between the two opposing strands of the DNA phosphate backbones when perceived from the minor groove side. This measure is informative of the intrinsic tendency of a DNA segment to show a widened or contracted minor groove. This approach was applied to a region of ±15 bp around the TBS (including the TBS) of all yeast promoters investigated in this study.

### Interface properties of TBP-containing complexes

The atomic coordinates of TBP in complex with Taf1p (4B0A)^54^, Mot1p (3OC3)^50^ and TFIIA (1RM1) were obtained from the Protein Data Bank (PDB). The PDB files were processed to only include TBP and the interaction partner of interest. For the complexes of TBP with Taf1p and Mot1p (both monomeric proteins) this was already done. However, the structure of TBP in complex with TFIIA also hosts DNA, which was first removed. The interfaces of TBP with the protein or DNA interaction partners were then ‘repaired' using FoldX 3.0 (http://foldxsuite.crg.eu/) with standard parameters, putting the structures of the complexes on the same energetic ‘footing' important for comparisons. The atomic contacts between TBP and its interaction partners were then characterized with the internal module of the Chimera software (https://www.cgl.ucsf.edu/chimera/). Furthermore, the accessible surface area of TBP and the buried surface area on complex formation was calculated using the Hotregions webserver^66^, which employs naccess (http://www.bioinf.manchester.ac.uk/naccess/) internally. Finally, for the different complexes containing TBP, the energy contributions of the interfaces were quantitatively estimated at the residue level using FoldX 3.0 with standard parameters.

### Turnover as a measure of dynamics of TBP at the promoter

TBP turnover data at 542 gene promoters were obtained from van Werven *et al.*^28^ TBP turnover is defined as the rate at which a new molecule of TBP binds at the promoter after an old one has been displaced.

### Testing for statistical significance

Statistical analysis was done using the R statistical package. Statistical significance was assessed using the Wilcoxon rank-sum test when comparing distributions and the *χ*^2^ test when comparing enrichments. The Mann–Whitney test (or Wilcoxon rank-sum test) was used to assess whether two samples were from the same population. It is a non-parametric test and does not assume a defined distribution. Statistical tests were corrected for multiple testing using the Benjamini and Hochberg method. The *χ*^2^ test for goodness-of-fit compares observed ratios with expected ratios for nominal scale data. It helps to assess whether there are significant differences between the expected frequencies and observed frequencies in one or more categories.

### Visualization of distributions

Distributions were represented by box plots, which highlight informative statics. The median value for each sample is shown with a horizontal black line. Boxes enclose values between the first and third quartile. The interquartile range (IQR) is calculated by subtracting the first quartile from the third quartile. All values that are 1.5 × IQR lower than the first quartile or 1.5 × IQR greater than the third quartile are considered to be outliers and were removed only from the figures to improve visualization.

### Markov chain modelling of promoter states and noise

Discrete time stochastic modelling of gene expression was performed to determine the impact of affinity, competition and residence time of TBP on noise. Markov chains (MCs) were used to model a graph based on our findings with five distinct microstates: free promoter (f), TBP:TBS (T), TBS:TBP:Mot1p (M), TBS:TBP:SAGA (S) and TBS:TBP:TFIID (D). The transition probabilities to switch between the microstates were chosen to be reflective of the cellular conditions in yeast cells. Every simulation was conducted for 150 time points and for 500 cells (see Supplementary Methods for more details and parameter selection). We did not explicitly model degradation. At the end of the simulation, the total expression level per cell and the variation thereof in the simulated population of cells was quantified using the CV. MCs were computed using the ‘markovchain' R library.

### Deletion of Spt3 and generation of the GFP tagged strains

We deleted the *SPT3* subunit of the SAGA complex from the *MATα* haploid Yeast strain Y6545 using nourseothricin (*Nat*) resistance plasmid pAG35 (ref 67). Synthetic genetic array technique was performed between ΔSpt3::Nat^r^ against the GFP collection (::*HIS3*; the library was a kind gift from J. Weissman, University of California, San Francisco, San Francisco, CA; Mating was performed on rich media plates, and selection for diploid cells was performed on plates with clonNAT Nourseothricin (Werner) and lacking *HIS*. Sporulation was then induced by transferring cells to nitrogen starvation plates for 5 days. Haploid cells containing all desired mutations were selected by transferring cells to plates containing all selection markers alongside the toxic amino-acid derivatives Canavanine and Thialysine (Sigma-Aldrich) to select against remaining diploids and lacking Leucine to select for only spores with an ‘a' mating type (Cohen and Schuldiner^68^). Synthetic genetic array procedure was validated by inspecting representative strains for the presence of the GFP-tagged strains and for the deletion of *SPT3* by PCR. To manipulate the collection in high-density format (384), we used a RoToR bench top colony arrayer (Singer Instruments).

### Protocol for flow cytometry

Wild type (WT) and *SPT3* deleted GFP-tagged yeast strains (see details below) were measured using flow cytometry. The comparison between the fluorescence emitted by wild type GFP-tagged strain (with *SPT3*) and the knockout shows the impact of the deletion. To process the cytometry data, the protocols from Newman *et al.*^7^, Weinberger *et al.*^13^ and Hornung *et al.*^69^ were followed. Cells were incubated in YPD medium at 30 °C overnight to stationary phase, then diluted to an optical density (O.D.) of 0.01 before growing for another 5–6 h prior to the measurement. An LSRII flow cytometer to measure fluorescence in standard mode at a velocity of 1–1.5 μl s^−1^ was used. GFP was excited at 488 nm and the fluorescence was collected through a 505-nm long-pass filter and 525-nm band-pass filter (Chroma Technology). Thousands of events were recorded from each well in the plate. The flow cytometry experiments were repeated in duplicates. The processing of the raw data was performed as reported before^13^. First, it consisted of filtering observations with extreme forward scattering values (0<SSC−A<218−1 and 0<FSC-A<218-1), and times of data collection. Then the measurements in the top and bottom 5% in terms of the scattering measured were discarded. To identify the subpopulation of small cells that did not bud, the measurements were gated to have a total scattering (SSC-A × FSC) below a quintile cutoff value of 0.5. To correct for the effect of size on GFP fluorescence, a linear model (GFP∼FSC−A+SSC−A) was defined. The size-corrected GFP values were obtained dividing the square of the raw GFP fluorescence measurements by the fitted values of the linear regression. The mean of the residuals of the linear regression indicate the s.d. of the measurements. The noise or CV was calculated with the corrected values dividing the s.d. by the mean of GFP fluorescence. To estimate the reproducibility of the measurement, the two replicates for the wild type, and the *SPT3* knockout were averaged and the s.e. was estimated.

## Additional information

**How to cite this article:** Ravarani, C. N. J. *et al.* Affinity and competition for TBP are molecular determinants of gene expression noise. *Nat. Commun.* 7:10417 doi: 10.1038/ncomms10417 (2016).

## Supplementary Material

Supplementary Information Supplementary Figures 1-7, Supplementary Notes 1-5, Supplementary Discussion, Supplementary Methods and Supplementary References

## Figures and Tables

**Figure 1:**
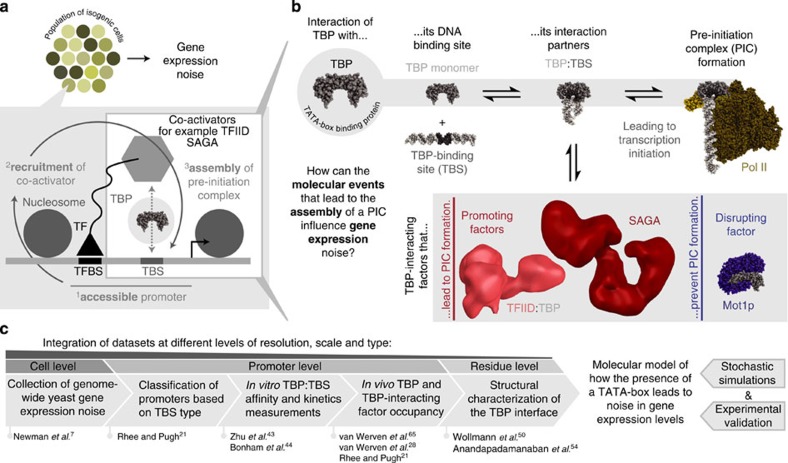
Framework for investigating how gene expression noise arises from the mechanistic details of the interaction of TBP with its partner proteins and their influence on PIC formation. (**a**) In a population of genetically identical cells (isogenic population), individual cells can show differences in their gene expression levels (shades of yellow). In order for genes to be expressed, their promoter needs to be accessible (nucleosome re-organization) and co-activating complexes need to be recruited (via transcription factors; TFs). (**b**) The TATA-box binding protein (TBP) is required for every transcriptional event in eukaryotic cells. TBP can exist in different functional assemblies. They are in dynamic equilibrium between a dimeric state and a monomeric state that in turn can form different TBP assemblies with (i) other general transcription factors (TFIIB and TFIIA in yellow), (ii) co-activators (TFIID in pink and SAGA in red) that promote pre-initiation complex (PIC) formation or (iii) disrupting factors (Mot1p in purple) that bind and evict the DNA bound TBP and prevent PIC formation. (**c**) To obtain mechanistic and molecular insights into the origins of noise, we integrated data from different levels of resolution, scales and types describing various aspects of transcription initiation in the yeast *Saccharomyces cerevisiae*, which were tested using stochastic simulations and were experimentally validated.
