# Supplementary material for: Affinity and competition for TBP are molecular determinants of gene expression noise
Source: Nat Commun. 2016 Feb 2;7:10417. doi: 10.1038/ncomms10417 (PMC4740812; doi:10.1038/ncomms10417)
Supplement: Supplementary Information — Supplementary Figures 1-7, Supplementary Notes 1-5, Supplementary Discussion, Supplementary Methods and Supplementary References [file ncomms10417-s1.pdf]

SUPPLEMENTARY FIGURES

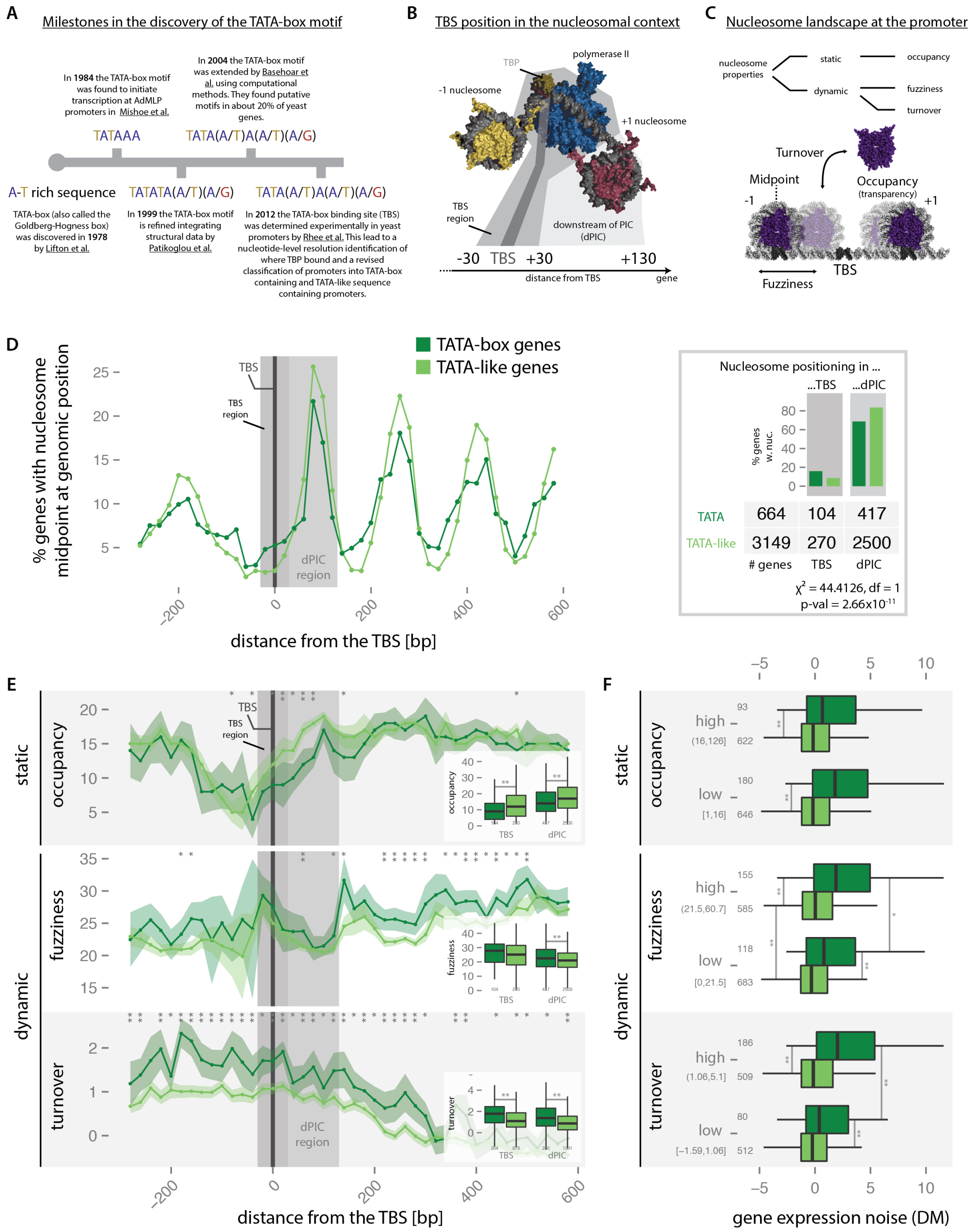

Supplementary Figure 1: Supplemental data for TBP interacting proteins that influence PIC formation and the framework for investigating the mechanistic details of the origins of noise. Related to Figure 1.
The evolution of the definition of the TATA-box motif was driven by the increased resolution of methods to study it. Binding of the TBS occurs in the nucleosomal context. Nucleosome dynamics are linked to gene expression noise. For further information about the plot content see Figure 1 in the main text.
(A) Discovery and changes to the definition of the TATA-box motif. The presence of A-T rich sequences in the promoter was first recognized in the 1970's and over the following decades the TATA-box motif is increasingly understood at nucleotide and atomic resolution[1]. Later this AT nucleotide enrichment was refined into a sequence motif found in the Adenovirus Major Late Promoter sequence (AdMLP) to which TBP binds[2]. Detailed structural characterization of TBP in complex with various TATA-box sequences with point mutations was used to further refine the motif[3]. Recent studies have considerably extended our knowledge on how the TATA-box operates on the genome-wide scale as opposed to a few highly studied promoters. In Basehoar et al. [4] the criteria used to call a functional TATA-box included that they are (i) a motif is computationally identifiable between –200 and –50 base pairs from the ATG start codon of the gene, (ii) conserved across different Saccharomyces species and (iii) associated with genes whose expression levels are particularly

sensitive to mutations in the DNA binding surface of TBP. This led them to describe the TATA-box with the following motif: TATA(A/T)A(A/T)(A/G). However this definition only indirectly takes into account whether TBP is physically bound at the TATA regions. This means that the TATA-boxes defined computationally remain putative with varying degree of confidence. Recently, TBP binding sites were defined at nucleotide resolution based on validated TBP binding events through ChIP-exo experiments by Rhee et al. This provides the most high-resolution view of TBP binding on a genomic-scale. In this work, this latest classification was used to group genes into two categories: those with a TATA-box motif and those with an experimentally verified TATA-like sequence. Furthermore we could now make use of the base-pair resolution information of TBP binding sites.

(B) The spatial organisation of factors at the promoter highlights the constraints imposed by the nucleosome landscape. The spatial organization highlights the region within which TBP has to identify the TBS to set the seed for PIC recruitment. Nucleosomes up and downstream of the TBS (–1 and +1 nucleosomes respectively) impose a barrier that has to be circumvented in order to recruit the Polymerase II. The two regions defined in our analysis are (i) the TBS region, which spans the TBS and the immediate neighborhood where the PIC forms and (ii) the region just downstream of where the polymerase binds (dPIC), which often includes the +1 nucleosome. The illustration is based on a model of the polymerase II in complex with TFIIB and TBP on a DNA template that was obtained from the Patrick Cramer group website (http://www.cramer.genzentrum.lmu.de/movies/) and nucleosomes were added up and downstream of the complex.

(C) Nucleosome static and dynamic properties. Nucleosomes have been intensely characterized at the biophysical level and high throughput studies have confirmed many of their properties on a genome-wide scale. Nucleosome properties can broadly be divided into two classes: (i) static properties such as nucleosome occupancy and nucleosome positioning and (ii) dynamic properties such as nucleosome turnover and nucleosome fuzziness. Each of these properties has functional implications on the nucleosome landscape at promoters in how they either facilitate or impair access of other factors to the DNA.

(D) The landscape of nucleosome occupancy around TBP binding sites. The midpoint positions of promoter nucleosomes are aligned to the TBS of yeast promoters, counted in bins of 20bp width, to be finally normalized to the total number of genes tested to give the percentage of genes with a nucleosome present at that position. The profile of TATA-box genes is given in dark green and the profile of TATA-like genes is given in light green. The inset (right) shows the sum of percentages of genes in the regions highlighted around the TBS in the main plot as bar plot (the TBS region and the dPIC region, see Supplementary Methods). The total number of genes belonging to the respective classes is given on the right. The nucleosomes in the respective regions for the different gene classes are furthermore displayed and a χ^2-test was performed to estimate significant differences between expected and observed occurrences.

(E) Static and dynamic nucleosome properties at promoters. The properties of the nucleosomes in the promoter were assessed, including occupancy (top), fuzziness (middle) and turnover (bottom). The different properties were calculated for the nucleosomes in the same 20bp bins in reference to the TBS (see panel D) and are displayed as the median values surrounded by a shaded area that represents box plot notches that are a useful guide to roughly estimate significant differences of median values. The properties are shown for TATA-box promoters (dark green) as well as for TATA-like promoters (light green). Furthermore the significance values for the difference in median at every bin were given based on a Wilcoxon rank-sum test that was corrected for multiple testing ("**" for $P < 0.01$ and "*" for $P < 0.05$). The plot insets at each nucleosome property consider all the nucleosomes in the TBS regions and PIC regions. The significance between the medians of TATA-box promoters vs. TATA-like promoters was estimated with Wilcoxon rank-sum tests. The number below the boxes indicates the number of nucleosomes in each class that were compared.

(F) The relationship between promoter nucleosome properties and gene expression noise. The different nucleosome properties of occupancy (top), fuzziness (middle) and turnover (bottom) were examined for their relationship with gene expression noise. Genes with nucleosomes in their TBS and PIC regions were included in the analysis. The nucleosome property values were averaged if more than one nucleosome was present in a gene's promoter. Finally the properties were split at their respective median to yield a high and low class for each. Gene expression noise distributions for genes with a TATA-box (dark green) or a TATA-like sequence (light green) in their promoter were plotted against the respective high and low property bins. The significance between the medians of TATA-box promoters vs. TATA-like promoters was again estimated with Wilcoxon rank-sum tests that were corrected for multiple testing ("**" for $P < 0.01$ and "*" for $P < 0.05$). The number on the left of the boxes indicates the number of genes with noise value in each class that was compared. The numbers under the high and low classes represent the ranges around the median at which the nucleosome properties were cut.

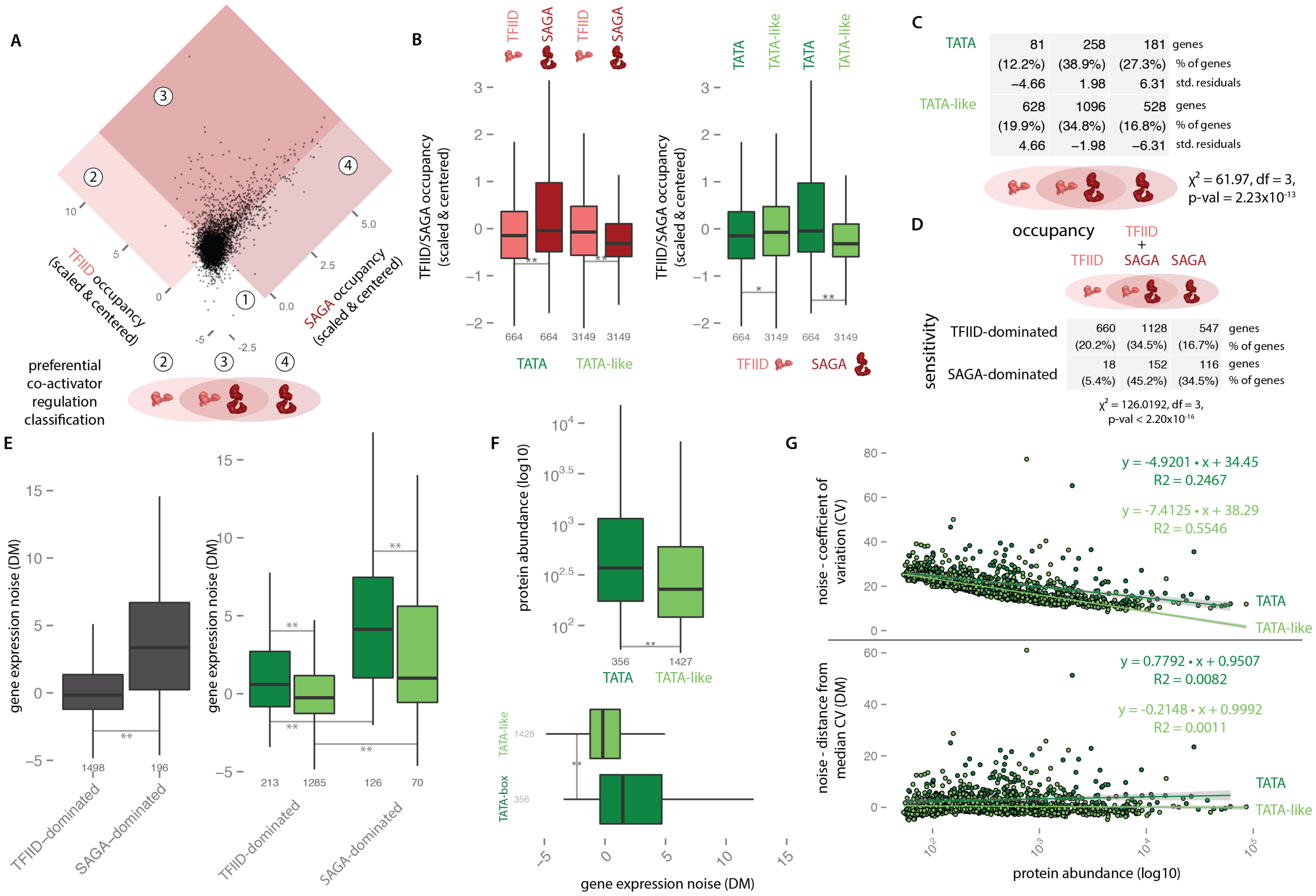

Supplementary Figure 2: Supplemental data for the Comparison and classification of TBS sequences and the relationship between TBS type, co-activator binding preference and gene expression noise. Related to Figure 2. Different TBP co-factors (SAGA and TFIID) show differences in preferential binding at promoters hosting different TBS sequences. Comparison between the occupancy-based classification and a previously published classification that is based on yeast strains where either co-activator was deleted and the effect on transcriptional output of genes was measured. For further information about the plot content see Figure 2 in the main text.

(A) Assignment of classification of promoters to different regulatory classes based on promoter occupancy of co-activators. Occupancy values of the TFIID complex (Taf1p) and the SAGA complex at all different yeast promoters are plotted against each other after their respective values were scaled by $\log_{10}$ and centered on the mean. The regions (different shades of red to pink) are defined based on the respective median occupancy values of TFIID and SAGA: high SAGA and TFIID occupancy (region 3), low SAGA and high TFIID occupancy (region 2) and high SAGA and low TFIID occupancy (region 4). The region of low SAGA and low TFIID occupancy (region 1) was not considered in the analysis as it mainly contained non-expressed genes, which is expected in the absence of co-activator complexes.

(B) TFIID and SAGA occupancy in TATA-box promoters and TATA-like promoters. Genes with either a TATA-box (dark green) or a TATA-like sequence (light green) in their promoter were compared for their occupancy of TFIID (pink) and SAGA (red). Their respective distributions are represented in a series of box plots. The left panel compares occupancy of the TFIID and SAGA co-activators with respect to the different TBS types. The right panel compares the occupancy of the different TBS types with respect to two co-factors TFIID and SAGA. At TATA-box containing promoters, the normalized occupancy of SAGA is higher than that of TFIID [5,6]. In contrast, at promoters containing a TATA-like sequence, the normalized occupancy of TFIID is higher than the one of SAGA (left). Among the promoters bound by TFIID, we find that TFIID occupancy is similar but slightly higher at TATA-like promoters when compared to TATA-box promoters (right; same data different grouping for comparison). The occupancy of SAGA in turn is higher at TATA-box promoters (right).

(C) Number of TATA-box and TATA-like genes in the different co-activator regulation classes. Genes hosting a TATA-box (dark green) or a TATA-like sequence (light green) in their promoter have been assigned to either co-activator regulation class defined above (high SAGA and TFIID occupancy, high SAGA and low TFIID occupancy, and low SAGA and high TFIID occupancy; Venn diagram). The number of genes in each class defined by TBS type and co-activator regulation is displayed along with the percentage this represents of the total genes of the TBS type respectively (in brackets below). A χ^2-test was performed to estimate statistically significant differences in terms of observed and expected frequencies of genes in the respective categories. The reported standard residuals indicate a rough guide to identify those classes that promote the observed trend the most, with values above 2 and below -2 being significant. This shows that TFIID-regulated genes were predominantly associated with a TATA-like sequence in the promoter, while SAGA-regulated genes were more often associated with a TATA-box containing promoter. There was no enrichment for either TBS type at promoters bound by both TFIID and SAGA.

(D) Comparison of co-activator regulation class based on occupancy data and co- activator sensitivity data. The table indicates the number of genes that are classified into different groups based on the occupancy of co-activators at their promoters (top) and their sensitivity to expression levels upon deletion of co-activators (left). The percentages given in brackets indicate the fraction of genes of the

total TFIID-dominated and SAGA- dominated genes under the different co-activator occupancy classes. The co-activator sensitivity data was obtained from Huisinga et al. [7].

(E) Gene expression noise of genes with a TATA-box or TATA-like sequences under TFIID- and SAGA-regulation. The distribution of gene expression noise for genes that were assigned as TFIID- and SAGA-dominated by Huisinga et al. [7] are plotted (left). The distributions of gene expression noise for genes from the previous plot were further split based on the TBS sequence in their promoter (i.e. TATA-box in dark green and TATA-like sequence in light green) and are plotted (right). Wilcoxon rank-sum tests that were corrected for multiple testing were performed to assess statistical significance of the differences between medians ("**" for $P < 0.01$). The number of genes in each class is given on the bottom of the plot.

(F) Protein abundance and noise value distribution for TATA-box and TATA-like genes. Genes were classified according to their TBS type and protein abundance (top) and noise (bottom) value distributions are displayed as box plots. Statistical significance was assessed using the Wilcoxon rank-sum test ("**" for $P < 0.01$). The numbers on the bottom/left of the boxes indicates the number of genes included in the analysis.

(G) Scaling of gene expression noise metric with protein abundance. Protein abundance is plotted against the coefficient of variation (CV), which describes the mean abundance normalized to the standard deviation of gene expression levels between cells (top) and the distance from median CV (DM), which subtracts a running median to the CV after ranking genes according to their mean protein abundance (bottom). For both measures of noise, genes with a TATA-box in their promoter were plotted (dark green) as well as genes with a TATA-like sequence in their promoter (lighter green). A linear regression was performed for protein abundance vs. the different noise measures, CV and DM.

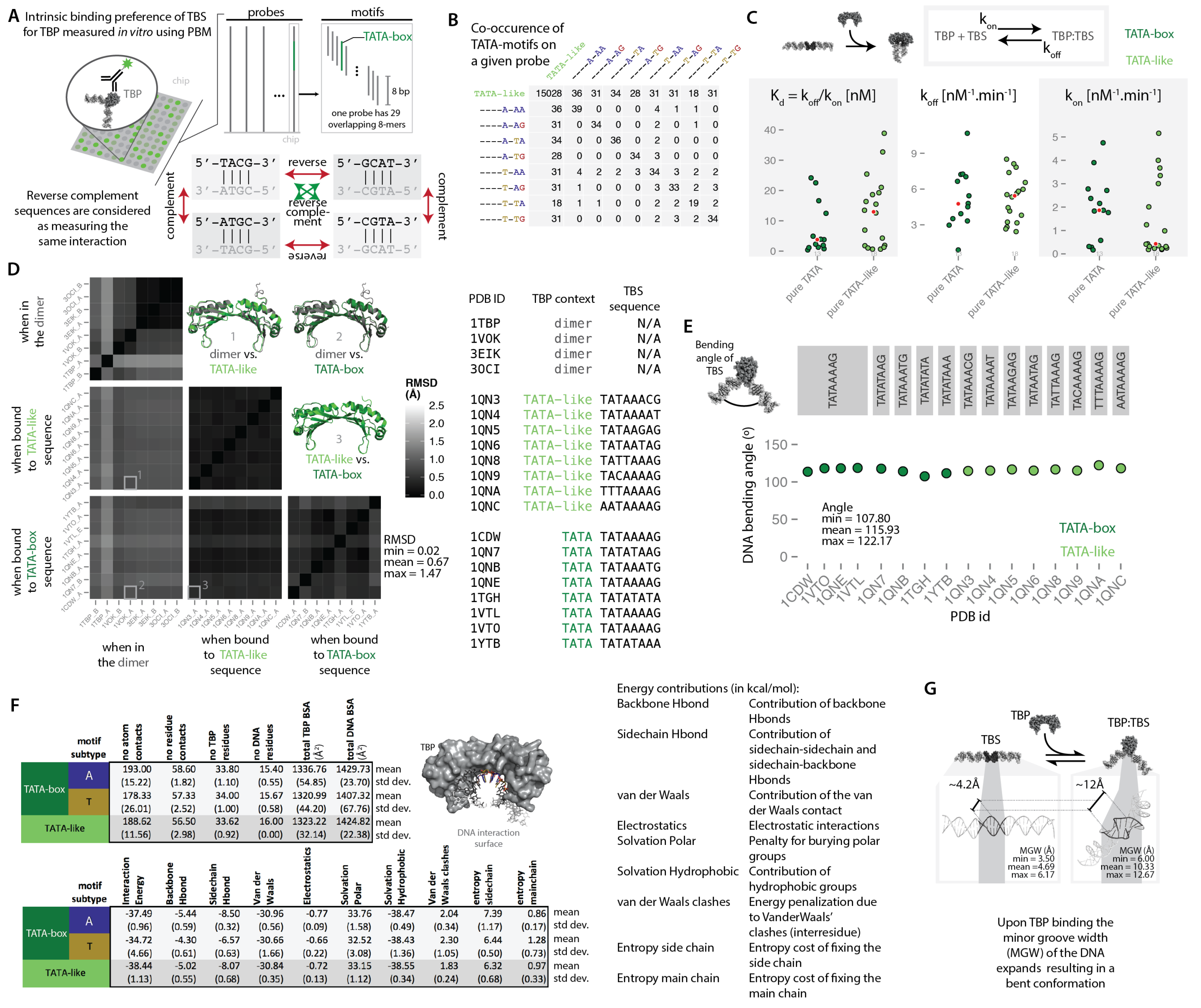

Supplementary Figure 3: Supplemental data for TBP binding affinity and intrinsic DNA flexibility of TBS sequences. Related to Figure 3.

Explanation of the design of the PBM microarray and the binding preference data that can be extracted from them. Comparison of kinetic and structural properties of TBS sequence types and their occurrence in vivo. For further information about the plot content see Figure 3 in the main text.

(A) Description of the PBM experiment assessing the intrinsic binding preference of monomeric TBP. The protein binding microarray (PBM) consist of a chip hosting double stranded DNA that covers the entire sequence space of 8-mers at multiple redundancies. Every probe on the chip has a length of 36 bp, which in an overlapping manner host a total of 29 sequences of length 8 (8-mers). Every 8-mer on all the probes of the chip are then classified as either being a TATA-box, a TATA-like or any other sequence. Binding of TBP to these different 8-mers is tested through the addition of TBP and quantified through the degree of fluorescence after incubation with a labeled antibody. The median value for each 8-mer across the different probes served as a surrogate measurement of intrinsic TBP binding affinity/preference for that motif. The PBM experiment cannot resolve measurements of binding for a sequence and its reverse complement (green arrows), hence probes with either motif or its reverse complement were considered as measuring the same binding event. Sequences that are either the reverse or the complement of each other (red arrows) can be distinguished as the direction of the phosphate backbone is different, and hence these sequences were considered as measuring different binding events.

(B) Frequencies of co-occurrence of motifs of different TBS assignment on the same probe. The nature of the design of the PBM chips has the advantage of representing the complete sequence space of 8-mers at high redundancy on a compact chip, however at the expense of having multiple 8-mers being potentially responsible for the binding signal observed. To deconvolute the measurements, the authors have chosen a strategy to take the median of the signal of all the probes that host the given 8-mer, considering that it occurred regularly on a different probe [8]. In this way they could establish the robustness of their method. However, to get a better assessment of co-occurrences of TATA-box and TATA-like sequences on the same probe, we quantified the co-occurrences of these classes of motifs in an all-against-all manner. The TATA-like sequences were grouped together for this purpose and the TATA-boxes are shown individually with the dashes representing the common portion of the motifs (TATA-A--).

(C) TBP binding kinetics to different TBS sequences. The rates of TBP binding to the TBS (k_{on}) and the rates of falling off again (k_{off}) have been measured on a PBM developed by Bohman et al. [9] to obtain dissociation constants (K_d). Probes with only a TATA-box sequence

(dark green dots) and probes with only a TATA-like sequence (light green dots) are plotted for K_d (left), k_{off} rates (middle) and k_{on} rates (right). The red dots for each class represent the respective median values. While the median "on" rate is higher for TATA-box compared to TATA-like TBS sequence, a Wilcoxon rank-sum test showed that the p-values for the differences are not statistically significant.
(D) TBP conformation remains constant when not bound to DNA, or bound to TATA-box/TATA-like sequences. The heatmap shows the RMSD values (in a grey gradient) comparing TBP structures in different contexts: in dimer form, when bound to TATA-like sequences and when bound to TATA-box sequences. The structures are highly similar as indicated by the low RMSD values (this is despite the taxonomic diversity of the structures). The structures were overlaid with Pymol to highlight the low RMSD between the TBP in the different contexts. The table on the right indicates the different TBS motifs that were crystallized in the different PDB entries. The results show that TBP remains structurally very similar and rigid in the free form or when bound to TATA-box/TATA-like DNA sequences.
(E) TBP bends TATA-box and TATA-like sequences to the same extent in crystal structures. The bending angles of TBS sequences were calculated between the position 1 and 8 of the motif that were connected by the motif dyad. For each position, i.e. position 1, the dyad and position 8, the phosphate atoms (PDB code "P") of opposite strands were used. This calculation reveals that irrespective of whether the TBS is a TATA-box or TATA-like sequence, it gets bent to the same extent in the final TBP:TBS complex.
(F) Residue-base interaction networks and interaction energies of the TBP:DNA interface. A variety of properties were calculated for the 16 structures of TBP in complex with a DNA segment. Together they help to characterize the TBP-DNA interface. The mean value for each property is provided for the different TBS types and TATA-box subtypes (dark green for TATA-boxes, yellow for the T_5 subset, blue for the A_5 subset and light green for TATA-like sequences) and the standard deviation is given below in brackets. The properties calculated include: the number of atomic contacts, the number of residue contacts, the number of TBP residues involved in the interaction, the number of DNA bases involved in the interaction, the total buried surface area of TBP, the total buried surface area of DNA and finally a set of energy contributions of the interaction calculated by FoldX 3.0 (a legend of the energy contributions is given at the bottom).
(G) Minor Groove Width (MGW) as an indicator of TBS bendability. Minor Groove Width (MGW) for different TBS structures and sequences. Upon binding of TBP, the MGW of the TBS widens dramatically leading to the bending of the DNA (left). The TBS is shown in dark grey in cartoon representation and the minimum, average and maximum MWMs (at the different positions within the DNA) are given for DNA in both the unbound and bound forms respectively (PDB: 1CDW and 1BNA)

```
A                                  TBP core
 24          TLQNIVSTVNLDCKLDLKAIALQARNAEYNPKRFAAVIMRIREPKTTALIFASGK   78  P28147   TBP1_ARATH
 66          TLQNIVATVTLGCRLDLKTVALHARNAEYNPKRFAAVIMRIREPKTTALIFASGK  120  P13393   TBP_YEAST
 24          TLQNVVATVNLSCKLDLKNIALRARNAEYNPKRFAAVIMRIREPKTTALIFASGK   78  Q8ST28   Q8ST28_ENCCU
             ****:*:**.* *:**** :**:********************************

 79     MVCTGAKSEDFSKMAARKYARIVQKLGFPAKFKDFKIQNIVGSCDVKFPIRLEGLAYSHA  138  P28147   TBP1_ARATH
121     MVVTGAKSEDDSKLASRKYARIIQKIGFAAKFTDFKIQNIVGSCDVKFPIRLEGLAFSHG  180  P13393   TBP_YEAST
 79     MVITGAKSEKSSRMAAQRYAKIIHKLGFNATFDDFKIQNIVSSCDIKFSIRLEGLAYAHS  138  Q8ST28   Q8ST28_ENCCU
        ** ******. *::*:::**:*::*:** *.* ********.***:** *******::*.

139     AFSSYEPELFPGLIYRMKVPKIVLLIFVSGKIVITGAKMRDETYKAFENIYPVLSE      194  P28147   TBP1_ARATH
181     TFSSYEPELFPGLIYRMVKPKIVLLIFVSGKIVLTGAKQREEIYQAFEAIYPVLSE      235  P13393   TBP_YEAST
139     NYCSYEPELFPGLIYRMVKPKIVLLIFVSGKIVLTGAKVRDDIYQAFNNIYPVLIQ      194  Q8ST28   Q8ST28_ENCCU
         :.**************  **************:**** *:: *:**: ***** :
```

Supplementary Figure 4: Supplemental data for properties of the TBP interaction interface with the different interaction partners. . Related to Figure 4.

Comparison of TBP sequences from diverse organisms show that TBP is highly conserved and can be used in comparative analyses. For further information about the plot content see Figure 4 in the main text.

(A) Sequence alignment of TBP crystallized in complex with different interaction partners. The TBP sequence of the crystallized core of the *Saccharomyces cerevisiae*, *Arabidopsis thaliana* and *Encephalitozoon cuniculi* structures are highly similar, aligning without insertions or deletions. In combination with structural alignments, they were used to compare the interaction surfaces between them and their respective interaction partners in the crystal structures. The alignment was performed on the UniProt website (http://www.uniprot.org/).

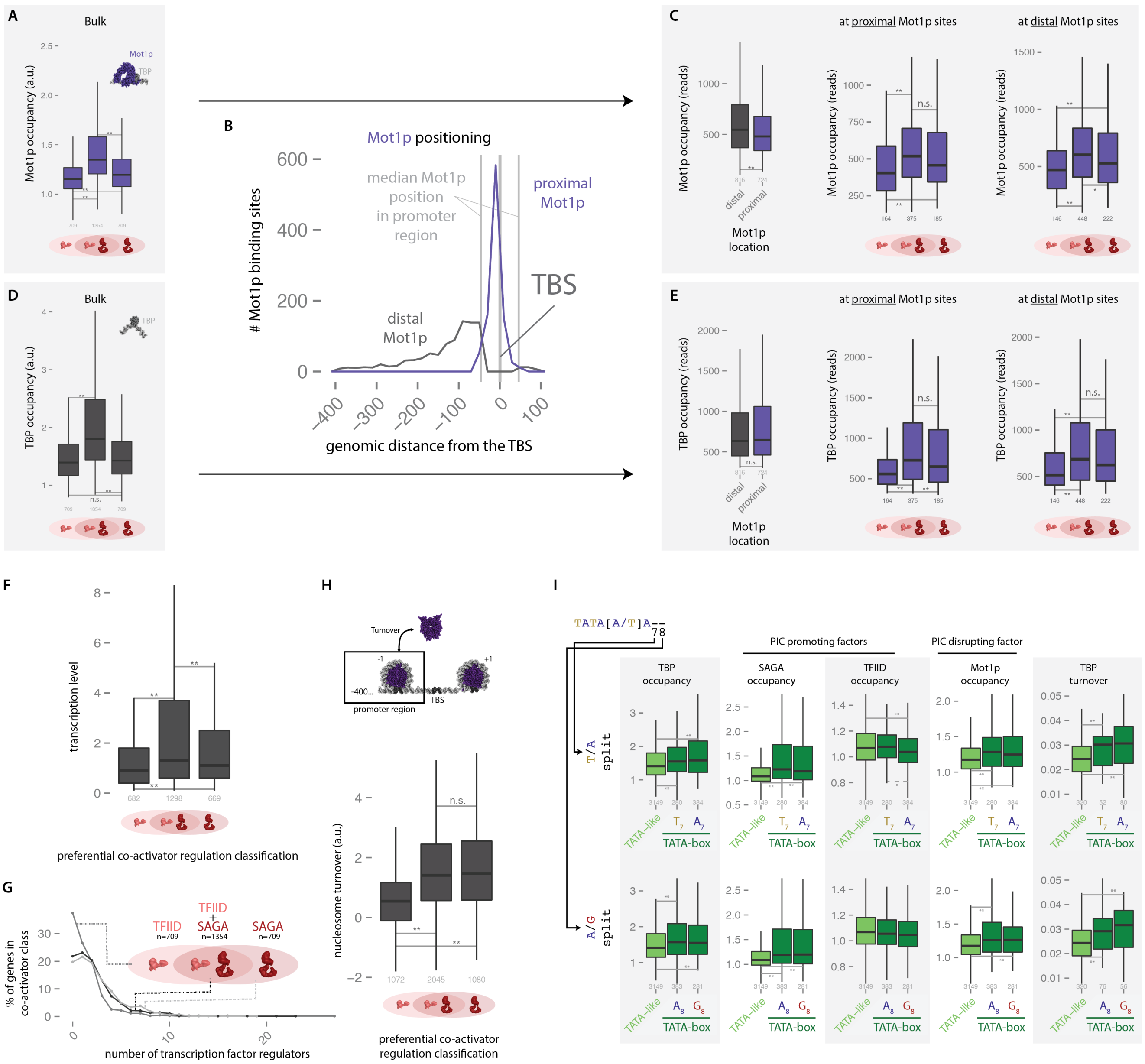

Supplementary Figure 5: Supplemental data for box plots of the distributions highlighting the relationship between TBS, TBP interacting proteins, TBP turnover and gene expression noise. Related to Figure 5.
The TATA-box sequence subtypes (A7 and T7) and (A8 and G8) do not affect the in vivo properties at yeast promoters. For further information about the plot content see Figure 5 in the main text.
(A) Mot1p occupancy at promoters regulated by different co-activators. The distributions of Mot1p occupancy, averaged across the whole promoter region (lower resolution measurements from ChIP-chip; on average ~250bp), for genes under different co-activator regulation classes are plotted. Statistical significance was assessed using the Wilcoxon rank-sum test ("**" for $P < 0.01$). The number below the boxes indicates the number of genes included in the analysis.
(B) Proximal and distal Mot1p binding sites. All Mot1p binding events were first assigned to the closest TBS. The proximal sites (purple) were differentiated from the distal sites (grey) by splitting them at the median (-46bp of the TBS) of all Mot1p sites.
(C) The distributions of Mot1p occupancy at proximal and distal sites. The occupancy of Mot1p at proximal and distal sites as measured by reads supporting the Mot1p binding event for distal (grey) and proximal sites (purple) is displayed in box plot on the left. The Mot1p occupancy distributions for genes belonging to the different co-activator regulation classes at proximal sites (middle) and distal sites are shown on the right.
(D) TBP occupancy at promoters. Since Mot1p does not bind to DNA directly and requires monomeric TBP, we investigated the TBP binding profile in promoter regions. Consistent with Mot1p's function, we observe that TBP occupancy is highest for genes regulated by both co-factors, suggesting that there are (possibly) non-functional TBP binding sites in the promoter region and Mot1p is required to evict TBP from such sites.
(E) The distributions of TBP occupancy at proximal and distal Mot1p binding sites. Distribution of TBP occupancy for the genes belonging to the different co-activator regulation classes at proximal sites (middle) and distal sites (right) are similar to the profiles of Mot1p occupancy.

(F) The expression levels are highest for genes regulated by both TFIID and SAGA. Transcription levels of genes classified as part of the different co-activator regulation were compared.
(G) TFIID/SAGA regulated genes have more of distinct TFs regulating them. The number of different transcription factors that regulate a gene are higher in genes regulated by either SAGA/TFIID or SAGA alone. The number of genes included in the analysis per class is given above the Venn diagram. The counts were normalized by the total number of all genes in a given co-activator class to obtain the percentage of genes (y-axis).
(H) Nucleosome turnover is higher at genes regulated by SAGA or TFIID and SAGA. Nucleosome turnover in the promoter regions upstream of TBSs (up to -400bp) is higher at genes regulated by both SAGA/TFIID and SAGA alone. This indicates that chromatin is more dynamic and that there is more opportunity for spurious TBP binding events in regions other than the functional TBS. Statistical significance for the differences between the distributions was assessed using the Wilcoxon rank-sum test ("**" for $P < 0.01$, and "*" for $P < 0.05$). The number below the boxes indicates the number of genes that were included in the analysis.
(I) TATA-box subtypes based on position 7 and 8 do not show differences in *in vivo* promoter properties. As a control, genes were again classified based on the TBS type in their promoter (dark green for TATA-box and light green for TATA-like sequences) however this time the TATA-box subtypes were defined based on position 7 (top row) and 8 (bottom row) of the motif. The distributions above are compared and Wilcoxon rank-sum tests that were corrected for multiple testing ("**" for $P < 0.01$, "*" for $P < 0.05$) were performed to assess statistically significant differences between the medians of the respective properties. The number of genes in each class is given below the plot.

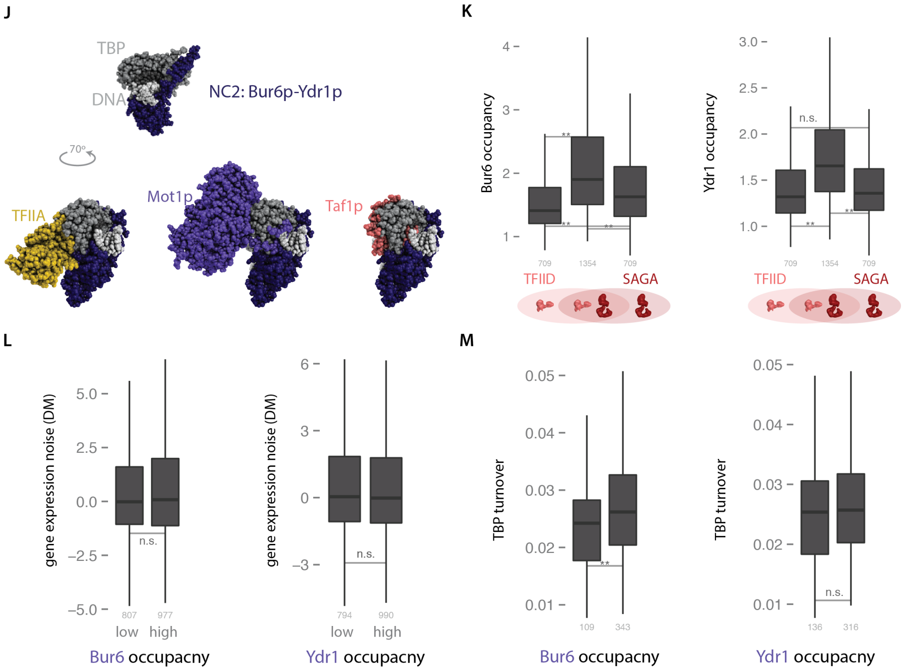

Supplementary Figure 5 (continued)

(J) Structural layout of NC2-TBP binding. Structural comparison between the TBP binding surface of NC2 (composed of Bur6p and Ydr1p), TFIIA (yellow), Mot1p (purple) and Taf1p (pink) of TFIID.
(K) NC2 occupancy at genes regulated by different co-activators. Occupancy of NC2 at genes preferentially regulated by TFIID, SAGA or both co-activators for the Bur6p subunit (left) and the Ydr1p subunit (right).
(L) NC2 occupancy vs. noise. Gene expression noise vs. NC2 occupancy levels split at their median (low and high occupancy representing below and above median) for the Bur6p subunit (left) and the Ydr1p subunit (right).
(M) NC2 occupancy vs. TBP turnover. TBP turnover vs. NC2 occupancy levels split at their median as in L. Statistical significance for the differences between the distributions was assessed using the Wilcoxon rank-sum test ("**" for $P < 0.01$, and "*" for $P < 0.05$). The number below the boxes indicates the number of genes that were included in the analysis.

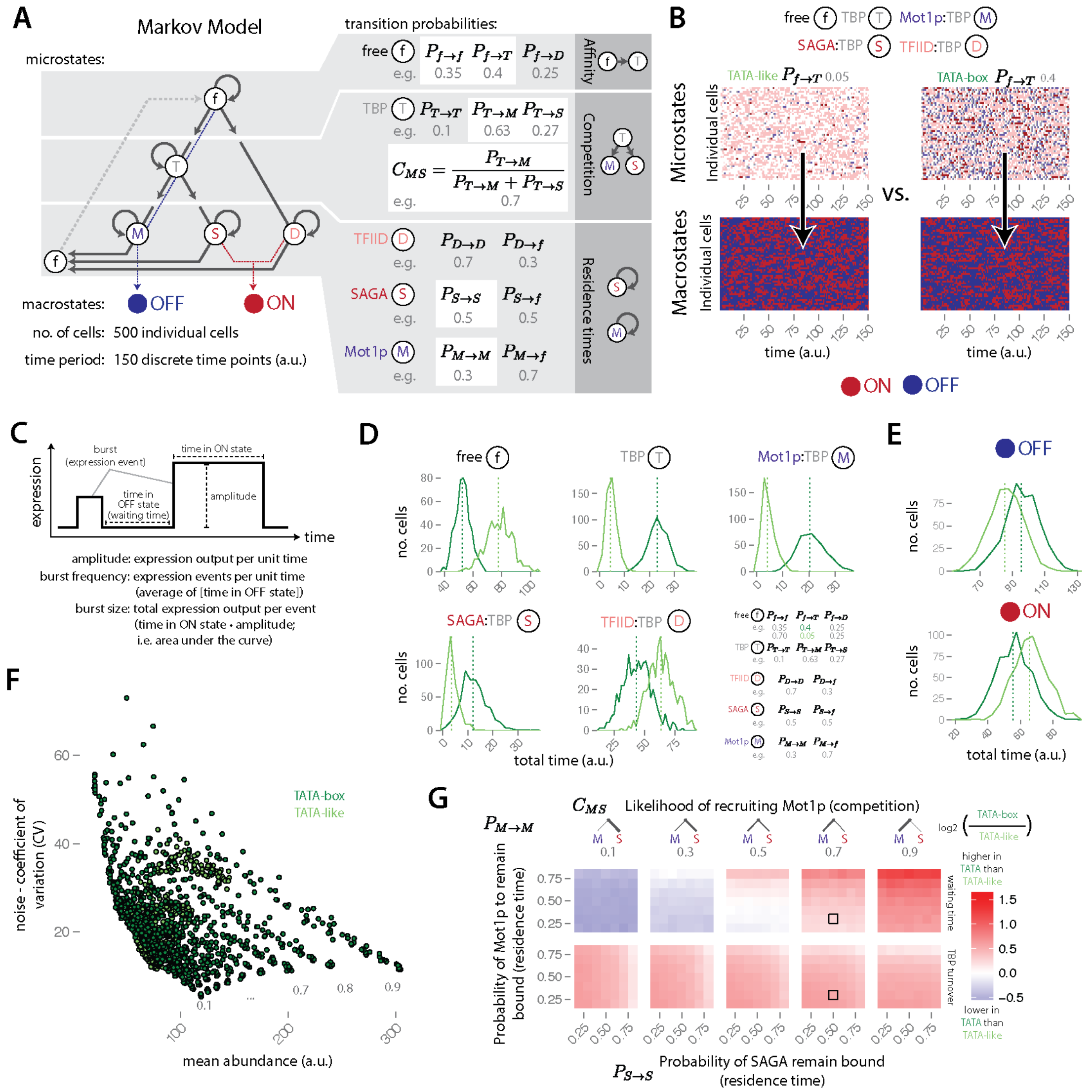

Supplementary Figure 6: Supplemental data for Stochastic simulation highlighting how TBP affinity for a TBS, competition between Mot1p and SAGA, and their residence times influence noise. Related to Figure 6.
Stochastic simulation reveals different regimes of gene expression noise that can be sampled by populations of cells. Modulating the extent of ON vs. OFF states at a promoter by the different TBP complexes influences gene expression noise. For further information about the plot content see Figure 6 in the main text.

(A) The possible TBP assemblies at the promoter (microstates) leading to potential transcriptional output (ON/OFF states). On the right, the transitions between the microstates were used to build a discrete-time Markov model. The transition probabilities to switch from one state to another or to remain in a state were assigned to reflect conditions in yeast cells (right; see **Supplementary Methods**). Together these probability values were used to model the intrinsic affinity of TBP for the TBS, the competition for the TBP:TBS complex by Mot1p and SAGA, and the residence times of Mot1p and SAGA. The model was used to simulate a cell population for 150 time points and for 500 individual cells. The transition probability parameters in the white/light gray boxes were varied over different ranges to test their effect on gene expression noise (the remaining variables were kept constant and apply to the other subpanels).

(B) Micro- and macrostate time traces for individual cells in the simulation. The TFIID:TBP and SAGA:TBP microstates were counted as ON macrostates and the free, TBP:TBS and Mot1p:TBP microstates were counted as OFF macrostates. The history of microstates for individual cells in a TATA-box or TATA-like promoter was modeled using the same parameters as in the main figure (top). The microstates were converted to their respective macrostates (bottom).

(C) Description of burst frequency and burst size as modeled in the simulations. Transcription initiation from a promoter is not a continuous process, but occurs in bursts. In other words, a promoter can either be in the ON state or the OFF state. A burst is defined as the time spent in the ON state, i.e. the period of time that transcripts are produced. Burst can be described with different parameters. For instance the time between bursts or the waiting times (time in OFF state) are related to the burst frequency, the number of bursts per unit of time. The total expression per burst is characterized by the burst size, which is the time spent in the ON state times the expression output per unit time (or amplitude).

(D) Distribution of the history of sampled microstates in TATA-box vs. TATA-like promoters. For each of the five possible microstates, the total number of time points (a.u.) that were sampled in a population of 500 cells over a time period of 150 (a.u.) are plotted as histograms and the respective mean values are indicated with a dashed line. The simulations between a TATA-box promoter and a

TATA-like promoter (dark green and light green) differ only in the $P_{f\text{->}T}$ probability parameter, which is related to the affinity of TBP to the TBS. The probability parameters that were used in the Markov model are given (right).
(E) Distribution of the history of sampled macrostates in TATA-box vs. TATA-like promoters. The distributions ON and OFF states of the simulated cell populations with a TATA-box and TATA-like promoter are plotted in a histogram and the respective mean values are indicated with a dashed line.
(F) Noise-mean relationship in TATA vs. TATA-like promoter simulations. The mean expression and noise in expression (CV) for every simulation (scanning a range of values for the various parameters) are plotted (TATA-box in dark green and TATA-like in light green). The negative scaling of noise (CV) with mean expression is observed in the simulations. The grey numbers indicate the variable SAGA residence times ($P_{S\text{->}S}$).
(G) Waiting times and TBP turnover comparison for TATA and TATA-like promoter simulations. The simulations were performed over the same range as in the main Figure 6C. The waiting time was calculated as the average time between ON macrostates (either through TFIID or SAGA) for each cell and then the population. The TBP turnover was calculated as the number of times the promoter assembly changed in the course of a simulation for each individual cell, which was then averaged over the population. The log2 was taken for the ratio between TATA-box (dark green) and TATA-like (light green) simulations (blue to red gradient). Black square boxes in the matrix highlight the parameter combination that was used to generate the simulation results shown in **Figure 6b.**

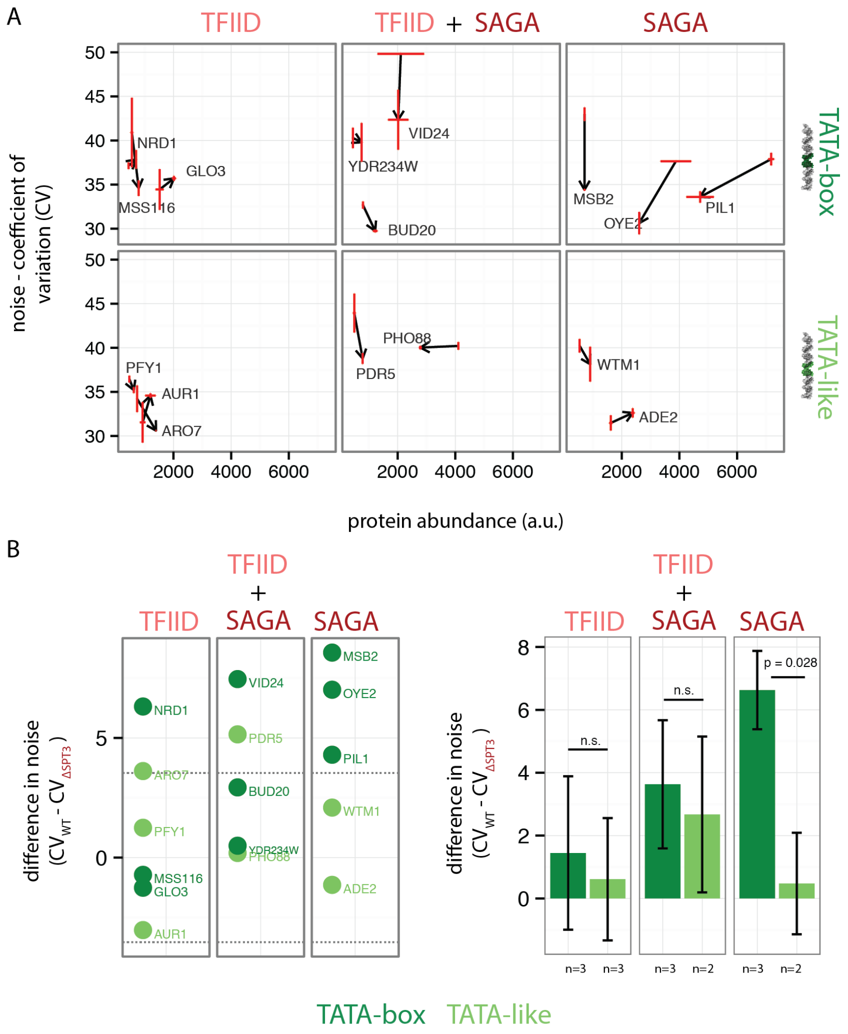

Supplementary Figure 7: Supplementary data for Deletion of SAGA impacts noise in the way predicted by the model. Related to Figure 7.

Experimental validation of important aspects of the model presented in this work. For further information about the plot content, see Figure 8 in the main text.

(A) The CV values from flow cytometry measurements of all 16 genes that were tested. The arrows indicate the direction of change of expression level and noise, starting from the WT and pointing towards the ΔSPT3 knockout (error bars in red).

(B) The effect of Δ SPT3 knockouts on noise. The difference in noise after deletion of SAGA's SPT3 subunit (CV_{WT} - $CV_{\Delta SPT3}$) for every gene individually (left) and aggregated into bar plots upon classification by TBS type and co-activator regulation (right). Error bars are indicated and p-values were obtained with a one-tailed Student's t-test. As to the agreement of our results with those of Raser and O'Shea[10], it is not easy to make a direct comparison for two reasons: Firstly, the authors delete a different subunit of SAGA with a very different function (i.e. Gcn5p, an acetyltransferase that does not compete with TBP binding but affect nucleosome organization), whereas we deleted the Spt3p subunit of SAGA, which competes with Mot1p to bind TBP. We chose Spt3p because we wanted to directly experimentally test our claim that competition for PIC formation influences noise. Secondly, the authors use a completely different experimental setup (i.e. induce the Pho5 gene by using 600mM Phytic acid as phosphate source). Under these conditions, the role of specific transcription factors may directly influence the strength of transcriptional initiation and gene expression noise. Please also see **Supplementary Discussion**.

<u>SUPPLEMENTARY NOTE 1</u>

Nucleosome landscapes differ at TBS promoter types

Binding of TBP to the DNA is the first step in forming the PIC, and hence transcription initiation. For this to be achieved, the TBP binding site (TBS) must be accessible. In eukaryotes, where most of the genome is wrapped by histone octamers into nucleosomes, this can pose a challenge. The intrinsic nucleosome landscape can be defined by static (occupancy) and dynamic (fuzziness and turnover) properties (see **Supplementary Fig. 1C**). Integration of these datasets allowed us to investigate whether TBS and the histones physically compete (or not) for the same site on the DNA in gene promoters. We first grouped genes according to their TBS type (TATA-box-containing promoters and TATA-like sequence-containing promoters) and then aligned the promoters according to their experimentally defined TBSs. Furthermore we defined two regions at and after the TBS (TBS region and dPIC region respectively, (see **Supplementary Fig. 1B**), in order to take into account spatial considerations required for the pre-initiation complex (PIC) formation and recruitment of the polymerase compared to the downstream of PIC formation (dPIC).

We first integrated this information with data on static nucleosome properties. Compared to the TATA-like promoters, TATA-box promoters have relatively more nucleosomes whose mid-point overlap with the TBS region, and fewer nucleosome midpoints in the dPIC region (see **Supplementary Fig. 1D**). However, the nucleosome occupancy is significantly lower in both regions for TATA-box promoters, when compared to the TATA-like promoters (see **Supplementary Fig. 1E**). This implies that at the TATA-box promoters, where nucleosome are more likely to overlap with the TBS (compared to TATA-like promoters), their lower occupancy values suggest that they are weakly populated. In absolute terms however, only around 20% of TATA-box genes have a nucleosome positioned at the TBS, with the remainder considered nucleosome free. Previous studies[11] have used the TSS as reference point when aligning different promoters and investigating the nucleosome architecture. Here we aligned promoters by the experimentally verified TBS binding sites to get a more accurate impression of the interplay between PIC formation and the nucleosome architecture.

A systematic analysis of dynamic nucleosome properties revealed interesting trends. In terms of the nucleosome fuzziness, while there was no significant difference in fuzziness in the TBS region, we observe an increase in fuzziness within the dPIC region for the TATA-box promoters compared to the TATA-like promoters. This difference in fuzziness was furthermore observed a further down in gene bodies (see **Supplementary Fig. 1E** middle panel). The most dramatic difference between the promoter classes was observed when one considers nucleosome turnover at both the TBS and dPIC regions. Nucleosomes that overlap with the regions where PIC forms have a higher turnover in the TATA-box promoters compared to the TATA-like promoters (see **Supplementary Fig. 1E** bottom panel). Thus it appears that in TATA-box promoters, nucleosomes that overlap with the PIC regions are not significantly shifted along the DNA (fuzziness) but instead are more often evicted from the DNA (turnover), thereby facilitating access to the TBS. These observations collectively suggest that the positioning and dynamics in the PIC-forming region significantly differ in TATA-box promoters and TATA-like promoters.

Dynamic nucleosome landscape is associated with noise

We then integrated the nucleosome landscape data with expression noise and found that the differences in nucleosome positioning that we observed between TATA-box promoters and TATA-like promoters were associated with distinct noise profiles. For each nucleosome property, we categorized genes into the High and Low groups based on the median values at the TBS for the nucleosome property investigated. We then analyzed the distributions of the values for the TATA and TATA-like promoters. In terms of nucleosome occupancy, irrespective of whether it was high or low, the noise of TATA-box genes was significantly higher than the TATA-like genes. However, we observed that there was little difference between the noise values for the two different occupancy groups for both promoter types (see **Supplementary Fig. 1F** top). Thus nucleosome occupancy does not appear to be correlated with noise. In terms of dynamic properties, we found that higher fuzziness in the nucleosome positioning at the TBS is associated with higher noise and this trend is irrespective of the promoter type. Nevertheless, irrespective of the nucleosome fuzziness level at the TBS, genes with TATA-box promoters had higher noise compared to those at the TATA-like promoters (see **Supplementary Fig. 1F** middle). In terms of nucleosome turnover at the PIC, we found that although genes with a TATA-box show higher noise than genes with a TATA-like sequence, we find that nucleosome turnover is associated with higher noise for TATA-box genes rather than TATA-like genes. In other words, genes with high nucleosome turnover and a TATA-box show more noise than the genes with low nucleosome turnover and a TATA-box (see **Supplementary Fig. 1F** bottom).

Taken together, these observations collectively suggest that higher nucleosome turnover, higher nucleosome fuzziness, lead to lower occupancy at the TBS of TATA-box promoters (see **Supplementary Fig. 1E** boxplot insets), which may result in competition between the histone octamer and PIC forming factors. This may result in mutually exclusive transcriptionally permissive and non-permissive states at a gene promoter that may be linked with gene expression noise (see **Supplementary Fig. 1F**). Although twice as many TATA genes have a nucleosome overlapping with their TBS, less than 20% of the genes

show this scenario. This suggests that factors other than nucleosome landscape may have an influence in generating noise (see **Supplementary Fig. 1D** right).

SUPPLEMENTARY NOTE 2

The role of intrinsic bendability of the TBS sequence in determining TBP assemblies

As it is the DNA that undergoes significant conformational change upon TBP binding, the intrinsic flexibility of certain DNA sequences in the unbound form may determine the affinity for TBP[12]. The more intrinsically flexible and dynamic the unbound DNA is, the lower is the cost of deformation (i.e. conformational strain) upon complex formation, which would enable easier formation of the TBP:TBS complex. In the unbound form, a stretch of DNA can sample a wide variety of conformations[13]. Being bendable increases the likelihood of forming the encounter complex with its interaction partner when available[14,15]. A number of studies have established that the DNA sequence is informative of flexibility[13,16,17] and correlates well with the protein-induced bendability as independently observed in crystal structures of protein-DNA complexes[17].

A hallmark of the TBP:TBS interaction is the extensive bending of the DNA in the final complex: TBP bends both TBS types by ~110° leading to broadening of the minor groove width (MGW) to ~12 Å (see **Supplementary Fig. 3G**). In this regard, the intrinsic MGW of the DNA in its unbound form is indicative of the extent to which the DNA in the unbound form is bendable and can be bent to form the final configuration (i.e. the TBP-bound form). This may possibly explain why the "off" rate of the complex is comparable, but the "on" rate to form the complex is higher for the TATA-box sequences and lower for the TATA-like sequences (i.e. the "on" rate is driven by the ability of the unbound DNA sequence to be flexible and easily bendable; **Supplementary Fig. 3C**). Furthermore, we find that for the T_5 subset, the relatively wide MGW extends throughout the whole motif, whereas there is a sharp drop in the A_5 set, possibly explaining the stronger binding of TBP to the T_5 subset (see **Fig. 3b**).

Compared to the TATA-like sequences, TATA-boxes are more bendable. This is because TATA-box sequences contain more A_PT base steps that show the weakest inter-base interactions[18]. Molecular Dynamics simulations have revealed that single point mutations within a TATA-box motif can have significant impact on the bendability of the motif, and can influence the energetic contribution required for TBP binding[18]. Thus, it is likely that the intrinsic structural flexibility of a TBS might play a significant role in determining the kinetics and thermodynamics of TBP binding[19]. How does TBP still bind to TATA-like sequences? It is likely that additional subunits of the TFIID complex indirectly facilitate TBP within the complex to bend the TBS. Direct interactions of TFIID subunits with neighboring elements (e.g. chromatin, transcriptional activators, etc.)[20,21] might keep the TBP proximal enough to interact with the low-affinity TATA-like sequences or alter the promoter DNA to facilitate the interaction[22]. This may explain why TFIID can bind both TATA-box and TATA-like sequences equally well. Thus, the intrinsic DNA structural properties of the TBS may explain the possible TBP assemblies that can be assembled at a promoter, i.e., whether TBP can bind with higher affinity independently and form the PIC via SAGA complex, or can be evicted by Mot1p, or can bind only as part of TFIID.

SUPPLEMENTARY NOTE 3

Mot1p occupancy at TFIID and SAGA regulated genes

The observation that Mot1p levels are higher at gene promoters where TFIID/SAGA jointly occupy may at first glance appear to be contradictory in nature. The following analyses provide possible explanations for this observation.

Why is Mot1p occupancy higher in genes regulated by both cofactors?
For the genes that are jointly regulated by TFIID/SAGA, it is intuitive to expect Mot1p levels to be of intermediate abundance at the TBS where a PIC forms. However, it is important to note that the resolution at which occupancy is measured for TFIID, SAGA and Mot1p does not permit us to infer whether these binding events happen at the same TBS or not. This is because the experiments for factor occupancy by Van Werven and co-workers[5] were done using tiling arrays (on average ~250bp resolution), which interrogate a large part of the promoter region rather than provide nucleotide level resolution (see **Supplementary Fig. 5A**). Thus the Mot1p occupancy measured in these experiments may not reflect its occupancy at just the TBS, but the entire promoter region. Therefore we analyzed high-resolution dataset by Zentner and Henikoff[23], which provides base-pair level binding information on Mot1p. A detailed analysis of this dataset revealed that there are two major binding modes for Mot1p, which the low-resolution data could not have resolved. For instance, we observed that Mot1p does not only bind specifically at the TBS, but binds to several regions upstream of the TBS in the promoter region. We classified Mot1p binding close to the TBS as proximal and those far away in the promoter as distal sites (see **Supplementary Fig. 5B**).

Mot1p cannot bind to DNA by itself and can only occur at sites where TBP is bound, and accessible[23]. As Mot1p is required to evict non-functional TBP binding (i.e. non-PIC forming TBP binding events) on the genome, it is likely that monomeric TBP binding at distal sites could lead to the observed high Mot1p binding at distal regions of the promoter. Using this dataset, we observe that the Mot1p occupancy at TFIID/SAGA regulated genes at proximal sites is no longer higher, but is comparable to genes that are predominantly regulated by SAGA (p=0.120; not significant; Wilcoxon rank-sum test; **Supplementary Fig. 5C**; middle). The occupancy is in fact slightly higher at distal sites (p=0.019; Wilcoxon rank-sum test; **Supplementary Fig. 5C**; right). The reason why Mot1p is not the highest in SAGA regulated gene promoters (but still higher than TFIID regulated genes) may be due to the fact that Mot1p has to compete with SAGA to bind to TBP, thereby reducing its occupancy.

When we analyzed the extent of bulk TBP binding (i.e. using probes in the promoter region), we again find that TBP occupancy is the highest for genes regulated by both TFIID and SAGA (see **Supplementary Fig. 5D**). Importantly, the TBP binding levels at the proximal and distal sites where Mot1p binds are no different between the genes regulated by SAGA and TFIID/SAGA[23] (see **Supplementary Fig. 5E**). These calculations on the high-resolution Mot1p binding dataset suggest that Mot1p can bind at the TBS and also in other, distal regions in the promoter where TBP binds (probably, non-functional binding). Such Mot1p binding events might reflect the requirement to evict "non-functional" TBP binding on the genome[23], and could be a possible explanation for the observation.

Why is TBP and Mot1p occupancy higher for genes regulated by both co-factors?

This raises another question as to why TBP occupancy is higher and why one observes more (possibly, non-functional) TBP binding events in distal regions of the promoter away from the PIC forming TBS. In the remaining section, we aim to provide a data-driven explanation of why this might happen.

Genes regulated by TFIID/SAGA are more highly expressed than the other two groups of genes (see **Supplementary Fig. 5F**; data from Holstege et al.[24]). This means that in a cell population, more individuals are transcriptionally active at that gene. Thus, in ChIP experiments, such genes might appear to have higher occupancy when interrogating the occupancy of certain factors[25,26]. Consistent with their high expression, genes with both TFIID/SAGA regulation have more number of distinct transcription factors binding to their promoter (see **Supplementary Fig. 5G;** data from Jothi et al.[27]). This can increase the likelihood of (a) recruiting TBP to both functional and non-functional sites, with further action of TBP remodeling factor such as Mot1p/NC2 required to reposition them to the functional, PIC forming sites[28]) and (b) recruiting TBP remodeling factors, thereby increasing their occupancy in the promoter regions[29]. Finally, and perhaps most importantly, we also observe that nucleosome turnover at these genes are high (see **Supplementary Fig. 5H**; data from Rufiange et al.[30]). This indicates that over a period of time, more nucleosome-free DNA is likely to be available for spurious TBP binding sites (probably non-functional), that otherwise would not be available due to the presence of nucleosomes. This could possibly explain the observed TBP binding profiles in the promoters of genes regulated by the different co-factor classes.

The interplay between NC2 and gene expression noise

NC2 is a dimer that is made up of two different subunits (Bur6p and Ydr1p). Earlier studies have suggested that NC2 slides TBP on the DNA and repositions TBP to functional sites at a promoter[31]. Genome-wide occupancy measurements have revealed that Bur6p and Ydr1p show different occupancy profiles across the genome, making it difficult to interpret whether factor binding is functional or not[5]. Furthermore, a thorough comparison of the structures of TBP with TFIIA and TBP with NC2 show relatively little overlap in terms of the surface where the two TBP-interacting factors bind TBP. NC2 forms a clamp like structure around the DNA and TBP. Such architecture would prevent access by TFIID or other PIC promoting factors (see **Supplementary Fig. 5J**). An investigation of a datasets on NC2 binding (see **Supplementary Fig. 5K**, below; dataset from van Werven and co-workers[5]) reveals that NC2 occupancy is lowest in genes primarily regulated by TFIID, and higher in SAGA regulated genes. Furthermore, NC2 occupancy is not directly associated with gene expression noise (see **Supplementary Fig. 5L**). Finally we observe that TBP turnover is differently affected by the two NC2 subunits (see **Supplementary Fig. 5M**), possibly suggesting different roles that are difficult to resolve with the current datasets. Given that the NC2 subunit occupancy data is possibly confounded, future research can help interpret these observations better.

SUPPLEMENTARY NOTE 4

Stochastic simulations reveal the role of affinity, competition and residence time on noise

Integrating the observations from the biochemical, biophysical, structural, and genome-scale occupancy data we performed a stochastic simulations of transcriptional initiation from an accessible TBS to explore the role of (a) affinity of TBP to TBS sequences, (b) competition between TBP interaction partners (specifically varying whether Mot1p or SAGA preferentially assemble at a TBP:TBS complex with constant probability for TFIID assembly), and (c) variable residence time of the TBP

complexes (specifically, varying Mot1p and SAGA with constant TFIID residence time) on noise (see **Fig. 6a**). The simulation assumes the promoter context to be the same (i.e. nucleosome and TFBS organization are not modeled explicitly). This provides an opportunity to monitor how the different TBS types alone affect the different TBP complexes ("microstates") that can assemble at a promoter in individual cells, which in turn affects the transcriptional output ("ON/OFF macrostates"), leading to noise in a cell population (see **Fig. 6a; Supplementary Methods**). While the simulation does not allow us to make accurate predictions on how the system would behave if perturbed in an experimental setup (as the cell most likely has multiple ways of adjusting its regulation), it does provide qualitative insights in to how noise is influenced when the properties of only the TBS are altered.

TBP affinity for its TBS sets the stage for the emergence of noise
We modeled the effect of TBP affinity to different TBS sequences ($P_{f->T}$; affinity parameter) in situations that are reflective of the observations described above, i.e. Mot1p is more likely to outcompete SAGA (C_{MS}; competition parameter), longer residence times for TFIID and SAGA when assembled and short residence times for Mot1p ($P_{D->D} > P_{S->S} > P_{M->M}$; residence time parameter). The simulations revealed that for the same situation, noise increases as the probability to assemble monomeric TBP increases (see **Fig. 6b**). To reflect the preferential binding of monomeric TBP to TATA-box sequences compared to TATA-like sequences, we fixed appropriate probability values for $P_{f->T}$ based on the PBM data for our further analyses. A more comprehensive simulation that systematically varied the competition and residence times of Mot1p and SAGA revealed the landscape of noise values that are attainable (see **Fig. 6c**). The resulting phase diagrams can be interpreted as a single gene in different situations (e.g., different outcomes for Mot1p v/s SAGA competition, longer residence times for SAGA).

Variable residence times and switching between SAGA and Mot1p at the TBS leads to noise
We observe that waiting time between the transcriptional ON states is longer for TATA-box genes in situations when Mot1p has a bigger impact at a promoter (either by increased Mot1p residence time or when SAGA is poorly recruited; see **Supplementary Fig. 6F**). This waiting time is a consequence of rapid recycling of monomeric TBP more often due to Mot1p, resulting in higher TBP turnover (see **Supplementary Fig. 6F**). If we model comparable transcriptional output from SAGA/TFIID, we find that TATA-box genes show low abundance compared to TATA-like genes. However, TATA-genes are known to have higher expression and more responsive in different environmental conditions[4,32]. This could stem from either the PIC intrinsically re-assembling more efficiently or certain TFs facilitating efficient SAGA recruitment (see **Supplementary Methods**). When taking this into account, our simulations reveal that TATA-genes are noisier and have more abundant expression. Thus, in a cell population, TBP will rapidly cycle between the unbound form and the TBP:TBS complex (OFF state). The variability (between different individuals) in switching to the occasional ON state with large expression burst via SAGA leads to differences in the expression output, resulting in higher noise.

Competition between TBP interacting proteins can influence noise
We observe that in many situations, noise is higher for TATA-box genes (see **Fig. 6c**). Surprisingly, the simulation also reveals the existence of other situations where TATA-box genes can have lower noise than TATA-like genes. For instance, in situations where SAGA outcompetes Mot1p (e.g. under low abundance of Mot1p and/or high abundance of SAGA), a TATA-box gene is less noisy (since SAGA more often wins the competition and/or residence time will be high). This can also happen when a strong transcriptional activator (e.g. Gal4p, interaction with Tra1p subunit of SAGA[33]) recruits and tethers SAGA to the promoter. In such situations, the influence of Mot1p is minimized and the promoter is more often in the ON state in TATA-box genes, thereby decreasing noise.

Residence time of Mot1p
The competition parameter reflects who is more likely to be recruited at a promoter (e.g. when abundance of one protein is higher than the other). The residence time represents how long they are likely to be retained at a promoter (e.g. when certain TFs can strongly interact with subunits of co-activator complexes and retain them at the promoter for longer periods of time). Thus the probability to remain bound at a promoter can be considered as a form of residence time if integrated over a time period. Independent of this, the Mot1p:TBP:TBS complex should indeed be transient as the enzymatic activity of Mot1p is typically high in the presence of cellular ATP[34]. Therefore, Mot1p should have low residence times in general. Thus, in the stochastic simulations, we modeled the Mot1p:TBP:TBS complex as the one with the lowest "residence time" ($P_{M->M}$ is set to only 0.3). We nevertheless chose Mot1p residence time as a variable, as it is useful to investigate the possible behavior of the TFIID and SAGA co-activator complexes and their impact on noise in the context of varying Mot1p residence times. TFIID and SAGA complexes have a certain probability to remain bound at the promoter, which is related to their modeled residence times. This can be modulated in a gene specific manner by certain transcription factors that help in more efficient recruitment of a co-activator and increase their residence times at a promoter (e.g. SAGA recruitment by Gal4p[33]). Likewise, there might be situations where Mot1p might be retained at a promoter for longer periods of time; for example when certain TFs might tether Mot1p at a promoter (i.e. increasing residence time of Mot1p) through direct interactions as suggested by proteomics experiments[29]. Thus to apply the same measure for all complexes under investigation, we used the same metric (residence time) for Mot1p as well.

SUPPLEMENTARY NOTE 5

Experimental validation of the model
We used CV as a measure of noise of a gene while comparing the wild type and SAGA mutant strains. Although the number of genes tested does not permit us to compute the DM values as described in Newman et al, it is valid to compare CV values specially while comparing the same gene in different genetic backgrounds. CV is expected to increase as abundance decreases (**Supplementary Fig. 2G**). Contrary to expectation, we found that for the genes with a TATA box (in the SAGA mutants), CV decreases as abundance decreases (**Supplementary Fig. 7A**). This suggests that cell-to-cell variability is significantly reduced compared to what is expected.

While the experimental results are consistent with the model, we are aware that we do not prove every aspect that the model can predict. We are also aware that the model is only an approximation of what might happen in the promoter of a gene. The extent of chromatin regulation, nucleosome context and TF binding may influence any of the steps in the model in a gene specific manner and hence can modulate transcription initiation and noise. This might explain why for genes regulated by both factors or TDIID alone, we find some genes where the observation is different from what one would expect. It would be interesting to investigate this further as it could highlight additional mechanisms that could be exploited to modulate noise. For the TATA-box/TFIID+SAGA genes, the prediction that the model actually makes is that the noise should not increase. This means that the noise can remain the same or can decrease. The choice for this depends on what extent TFIID can take over the regulation of the gene in the absence of SAGA. Thus the extent of occupancy of TFIID in the SAGA knockout cells could shed more light on this issue. This in turn could depend on the promoter architecture of the gene. Finally, we note that the model predicts many more scenarios than what we could possibly test in this study, and thus points to novel testable hypothesis that can be examined through further experiments. For instance, altering TBP affinity at a TBS, or Mot1p levels may influence TBP turnover, and hence noise in ways that could be predicted by our model.

SUPPLEMENTARY DISCUSSION

Factors other than TBP can influence gene expression noise
While reported observations are global trends and consistent with a number of genes, all trends reported here do not need to and are unlikely to apply to every gene. A number of additional factors can influence (or over-ride) the TBP complexes at a promoter: the residence time, access to the promoter, turnover rates and multiple neighboring initiation sites. The nature of the promoter sequence, such as the strength of the binding site for specific TFs, rigidity, or the ability to form or disrupt chromatin, can all influence PIC assembly, residence time, the exact initiation site and turnover rates[35-39]. Unstructured activation domains of TFs can recruit TBP, GTFs, or SAGA or TFIID directly[40]. This may explain why despite the preference for TBP to bind specific TBS, the presence of certain TF binding sites can influence the co-activator assembly in a promoter[33,41,42]. More fundamentally, nucleosome dynamics[11,43], nucleosome positioning[44], chromatin modification status, and chromatin factors[45] can all influence the access of TBP to the TBS and may determine the extent of variation in gene expression from a given promoter. Transcriptional poising at a promoter, transcriptional elongation rates, abortive transcription initiation[46], and the availability of factors such as NELF/DSIF that influence transcript synthesis rates could also contribute to gene specific variation in expression levels. Furthermore, in addition to transcription, gene expression stochasticity can be amplified or buffered at the post-transcriptional, translational, or post-translational level in a gene-specific or a pathway specific manner[47,48].

The model as a starting point to consider additional players that influence noise
The model serves as a starting point to consider the role of additional factors and homologs in the transcriptional machinery. It can be used to interpret the effect of non-coding SNPs and the effect of naturally occurring mutations in the promoter regions on gene expression stochasticity[49-51]. The principles described here can be exploited in synthetic biology applications that are aimed at tuning the level of stochastic gene expression. Another implication is that in addition to alterations in the expression level of TBP or mutations in the TBS, variation in the expression level of TBP interacting proteins will globally affect noise by redistributing the abundance of distinct TBP complexes and should therefore be considered as global regulators of noise. Indeed, temperature sensitive inactivation of Mot1p resulted in preferential accumulation of TBP at TATA-box containing promoters, leading to their increased expression[23]. Thus, such proteins might be attractive targets to globally regulate noise and to push the cell population to either have consistent or variable level of gene expression. Such a strategy could be exploited to counter the massive variability in gene expression levels seen in cancer cell populations[52-54] and other single celled eukaryotic pathogens[55]. Such a strategy can also be exploited to aid reprogramming of stem cells[56]. Finally, we suggest that the interplay between affinity and competition for an essential regulatory factor can drive distinct assemblies between individuals in a cell population and lead to heterogeneities in protein assemblies, resulting in expression variability. This principle may represent a more general framework that is applicable to every major step along the process of gene expression.
The model allows for rationalization of previously published perturbation studies

Earlier experiments showed that introducing mutations in a TATA-box resulted in reduced gene expression noise as measured by CV[57,58]. The mutations introduced converted the TATA-box to a TATA-like sequence, which should affect monomeric TBP binding (inferred from PBM data), and thereby minimize noise. Other studies[59,60] have shown that removing or mutating a TATA-box to a TATA-like sequence resulted in lower mean expression while having a similar CV ($CV=\sigma/\mu$). Since the CV remained the same, it was inferred that there was little contribution to noise by a TATA-box. However, for the CV to stay constant and the mean expression (μ) to decrease, the spread (standard deviation, σ) also has to decrease in the TATA-box mutants. This in fact suggests that removing/mutating the TATA-box does minimize the variability in the expression level of a gene between individuals in a cell population. In other words, if the TATA-box did not have an influence on noise, for a decrease in μ, the CV should be higher according to the well-documented negative-scaling relationship[61,62] between CV and μ (CV increases as μ decreases; **Supplementary Fig. 2G**; see also figure 4B and figure 5 in Bar-Even et al.[34]). The observation that the CV remains the same upon mutating/removing the TATA-box (rather than increase) in fact seems to reinforce the view that the TBS does influence noise. In molecular terms, in the mutated promoter, monomeric TBP (and hence SAGA or Mot1p) may not be effectively assembled. This might result in less efficient assembly of SAGA but may not affect binding by TFIID, leading to lower mean expression. TFIID binding may result in lower, but stable and consistent transcriptional output, leading to less variability between individual cells that express the gene. In another perturbation study, upon deletion of SAGA subunits, Weinberger and co-workers demonstrated that the yeast strains showed lower CV values from several TATA-gene promoters[45]. The model can explain this observation as follows: SAGA removal results in TFIID based regulation (as TFIID can regulate TATA-box and TATA-like promoters equally well). This may lead to a stable PIC assembly in some individuals; hence lower (but consistent) transcriptional output, and low noise.

Implications of the findings
An important implication of our findings is that in addition to alterations in the expression level of TBP or mutations in the TBS, variation in the expression level of TBP interacting proteins (e.g. SAGA, Mot1p and NC2) will globally affect noise by redistributing the abundance of distinct TBP complexes and should therefore be considered as global regulators of noise. Thus, such proteins might be promising targets to globally regulate noise and to push the cell population to either have consistent or variable level of gene expression. Such a strategy could be exploited in synthetic biology applications, and to counter the massive variability in gene expression levels seen in cancer cell populations[52-54] and single celled eukaryotic pathogens[55]. It can also be exploited to aid reprogramming of stem cells[56].

SUPPLEMENTARY METHODS

Genome-wide dataset on gene expression noise. Gene expression noise data were obtained from Newman et al.[62]. In this study, the authors performed single cell fluorescence measurements on 4,159 GFP-tagged yeast strains after growing them to mid-log phase in rich (YEPD) medium, starting from single colonies, and obtained reproducible measurements for 2500 strains. Noise values for every gene were computed as the ratio of the standard deviation over the of mean fluorescence intensity for the entire cell population (coefficient of variation; CV). The total noise measured can be further divided into two contributing portions, i.e. extrinsic, which describes the cell-to-cell variability in genes expression that affects all the genes equally, and intrinsic noise which reflects the noise that is specific to a given gene[63,64]. To minimize the contribution of extrinsic noise to the total noise measured the growing cells used in the experiment were filtered to only include highly similar cells in terms of granularity and size. The filtering also revealed an inverse relationship between protein abundance and noise[61]. To account for this, the data was normalized to arrive at an abundance-independent measure of noise, called distance from the running median CV (DM). This measure is computed by calculating the distance to the median CV (over a window of 7 genes) after ordering the genes according to their abundance. We found 1,804 protein coding genes for which information on these variables was available, and for all calculations we used the DM values in YPD conditions as noise value.

TBS classification. We obtained the TBP-binding sequence (TBS) classification status (TATA-box or TATA-like) for 4231 mRNA coding genes from Rhee & Pugh[6]. The main improvement of the dataset over previous ones is the base pair resolution of localization of TBP on a genome-wide scale. This high resolution was achieved via a new technique termed ChIP-exo, whereby the DNA in the pulled-down chromatin is digested with lambda exonuclease from the 5' to 3' direction, trimming it right up to the cross-linking site of the DNA to the protein of interest. Next generation sequencing of this DNA material identifies the precise boundaries of the protein binding events. The high resolution of this method allowed the authors to discover that at every promoter with TFIIB occupancy, which requires TBP binding first, there is a TATA-box motif with up to two mismatches. This allowed for a reclassification of genes into either TATA-box genes if the motif was "pure", or TATA-like genes if their promoter hosts a motif with mismatches.

Classification of promoter regions around the TBS. In order to take into account spatial considerations for the pre-initiation complex (PIC) formation, we divided the promoter into two regions based on the location around the TBS of each gene (in contrast to previous studies that mostly used the transcription start sites). In this way we defined (i) the TBS region surrounding the TBS by 30 bp up- and downstream, describing the location and immediate surroundings of where TBP binds and the PIC forms, and (ii) the dPIC region from 30 bp downstream of the TBS to 130 bp downstream of the TBS, which describes the region immediately downstream of the PIC including the +1 nucleosome region.

Nucleosome midpoints, occupancy, fuzziness and turnover. The properties and dynamics of nucleosomes have been described by various measures that can broadly be classified into static properties, such as occupancy and positioning (mid-points) and dynamic properties such as turnover and fuzziness. All these measures are based on population experiments, but since a nucleosome cannot exist at the same genomic locus in the same cell via steric occlusion, the measures are indicative of the nucleosome landscape that exists at the promoter and of how it can influence access of the transcriptional machinery to the promoter. We extracted the nucleosome positions (midpoints), occupancy and fuzziness from Mavrich et al.[65]. The authors pulled down TAP-tagged H3 and H4 nucleosomes after cross-linking the chromatin, and performed an MNase digestion to trim the wrapped DNA to the histone core, i.e. the nucleosome boundaries. The DNA material was then released via reverse crosslinking and digestion of protein material and prepared for next-generation sequencing (NGS) before aligning the sequenced reads back to the yeast genome. From the genome-wide distribution of reads (with an offset of 73 bp to identify the midpoint of nucleosomes) peaks were called to identify nucleosome midpoints. The number of reads at these midpoints, i.e. the number of supporting reads, was interpreted as nucleosome occupancy, and the distribution of these reads was interpreted as nucleosome fuzziness (or sliding). A genome-wide nucleosome turnover dataset was obtained from Rufiange et al.[30]. Here the authors developed a yeast strain with one copy of histone H3 tagged with Myc (H3-Myc) under the endogenous promoter and one H3 copy under a galactose inducible promoter with a Flag-tag (H3-Flag). The cells were initially grown in raffinose medium before one part of the population was arrested in G1 phase via the addition of alpha factor, whereas the remaining population was shifted to galactose containing medium and let to grow for an hour. After growth, the cells were cross-linked and the histones were pulled down via their respective tags to extract the bound DNA segments. In this way the authors could extract replication independent nucleosome turnover rates when comparing the ratio of H3-Flag- to H3-Myc-occupied DNA at promoters on a genomic tile microarray. In case a gene hosted more than one nucleosome within these regions (or probes in the case of the nucleosome turnover data), the median value was taken respectively to represent the nucleosome property in the TBS and PIC regions.

Occupancy of TBP interaction partners in promoter regions. The genome-wide binding profiles of TBP, Mot1p, TFIID (Taf1p subunit), SAGA (Spt20p subunit), and Pol II across the entire yeast genome were acquired from van Werven and co-workers [5] who employed chromatin immunoprecipitation (ChIP)–chip using cells in their exponential growth phase. The

genomic probe enrichment at time point 0 was used and for each gene. The median factor occupancy was computed for probes situated in the promoter region.

Classification of genes according to the bound co-activators in their promoters. Every gene was classified according to whether it was TFIID regulated or SAGA regulated (**Supplementary Fig. 2A**). As an intuitive measure of regulation, genes that have an occupancy value above the median of all genes' promoters for a factor (Taf1p or Spt20p) are considered to be regulated by that factor. Genes with an occupancy value below the median are considered as not regulated by that factor. This classification for TFIID regulation and SAGA regulation respectively leads to four possible states for a promoter: TFIID and SAGA regulated (+/+), only TFIID regulated (+/-), only SAGA regulated (-/+) and neither TFIID nor SAGA regulated (-/-). Importantly, this data preprocessing leads to the proportions of 50% TFIID/SAGA genes and 25% solely TFIID and 25% solely SAGA (i.e., the independent classification of genes by the median occupancy of TFIID and SAGA respectively that lead to these proportions). These fixed proportions are the consequence of splitting on the median and may therefore not reflect true proportions. Finally rescaling and centering was applied for visual clarity and does not have an effect on calculating the respective median of SAGA and TFIID occupancy. We decided on splitting the data by the respective median occupancy of TFIID and SAGA, as we believe this is an intuitive way to classify the data when making the point that either cofactor or both predominantly occupy some promoters. To ensure that the key observations are independent of the way in which we classify the genes, we have also performed another quintile-based classification (tertile-classification where respective middle bins were removed) to split the data and found the results to be similar (data not shown). This suggests that the interpretation of the trend is not dependent on the approach we used for classifying genes. Genes where either factor could not be detected were excluded from the analysis. This classification (which is based on binding data for a factor) was further confirmed via a comparison with a dataset that evaluated the impact of co-activator knockouts on expression output to group them as TFIID-dependent genes or SAGA-dependent genes. The co-activator sensitivity data was obtained from Huisinga et al.[7]. This analysis showed consistent trends with the occupancy-based classification (**Supplementary Fig. 2D-E**). Upon classification of genes based on their co-activator presence, we investigated the nature of the TBS types in such promoters (TATA-box or TATA-like sequence).

Intrinsic TBP binding preference from PBM experiments. The raw data of the PBM chips was collected from the Bulyk group website (http://the_brain.bwh.harvard.edu/uniprobe/downloads.php). The authors have generated a microarray chip with a set of synthetic double stranded DNA sequences that together represent all possible 10-mers of DNA. To print all the (4^{10} = 1.049 million) sequences on a single chip of ~65,000 probes, the sequence was designed based on a de Bruijn sequence of order 10, which is a sequence where all possible 10 bp long subsequences exist in an overlapping fashion and are present only once. This unbiased set of sequences on the PBM chip can then be assessed for their protein binding levels, when incubating the chip with a GST-tagged protein. Binding of the protein to the DNA probes is then detected using a GFP-tagged antibody to the GST. The authors developed and used this method to determine the intrinsic binding preferences of various transcription factors and chromatin regulators[8,66]. Here a subset of this dataset that was collected for TBP was analyzed in order to investigate the intrinsic binding preference of the TBP for given DNA motifs. Because the DNA sequences on the PBM were designed with a de Bruijn sequence of order 10, all possible 8-mer sequences, the sequence length that is bound by TBP, are replicated on average 32 times (palindromic sequences 16 times). This redundancy of measurements provides essential information to estimate the intrinsic binding affinity reliably across probes. Thus, the fluorescence signal intensity for every probe reflects how much TBP is bound. Hence the raw data consists of a single fluorescence value associated to a DNA sequence probe on the chip that hosts 29 overlapping 8-mer motifs (see **Supplementary Fig. 3A**). To deconvolute and approximate, which one of the contained motifs is binding the TBP, the median value of the many replicate measurements of the same motif on different probes was calculated as this has been shown to be a good indicator[8,66]. Finally the per motif median signal computed in this way is informative of TBP's intrinsic binding preference to specific sequences. The motifs in the probes were then classified based on their TBS sequence types (TATA-box, TATA-like sequence and other 8-mers). As the chip could not distinguish between different orientations of binding events, the median signal of the probe values was computed for every 8-mer together with their reverse complement (see **Supplementary Fig. 3B**). As mentioned earlier, the TATA-box motif has the consensus: TATAWAWR (W is either A or T, R is either A or G). Since sequences and their reverse complements were considered together, it is possible that a TATA-like sequence can be considered a TATA-box sequence in the reverse complement orientation. In these cases all the motifs were assigned as a TATA-box. Although the median signal from the different probes hosting the given motif is a good indicator for the intrinsic preference of TBP to bind, how many times different TBS sequences co-occur on a probe on the chip are an important consideration (see **Supplementary Fig. 3C**). Only a minority of TBS sequences were found to co-occur on the same probe.

Intrinsic binding kinetics of TBP. The K_d, k_{on} and k_{off} rates of TBP for TATA-box sequences and TATA-like sequences were taken from Bonham et al.[9]. Here the authors printed a set of DNA sequences of various sources (e.g. yeast promoters, human promoters, other genomic sequences) onto a microarray and incubated them with TBP and other general transcription factors (TFIIA and TFIIB). In this way they could not only assess the affinity and specificity of TBP for the different sequences via equilibrium binding measurements, but they could also assess kinetics of binding and dissociation

(**Supplementary Fig. 3D**). The system they developed is based on a TIRF-PBM method (total internal reflection florescence) that can measure the binding of TBP using fluorescence in real-time, thus opening up the possibility to study the kinetics of TBP binding in a high throughput manner. Here we assigned probes into different classes based on their TBS sequence content; sequences that only contained a single TATA-box were considered as "pure TATA-box" probes, those with only TATA-like sequences were considered as "pure TATA-like" probes. For sequences without a TATA-box nor a TATA-like sequence, as well as probes hosting both TBS types, it would be impossible to deconvolute their respective contributions on the binding profiles and kinetics measurements, and hence were not considered in the analyses.

Comparison of TBP structures when bound to DNA. TBP structures either in the dimeric form[67-69] or bound to different DNA sequences[3 70-74] were compared to each other in terms of their RMSD in an all-vs-all manner. Since TBP in the different complexes comes from different organisms (e.g. *Saccharomyces cerevisiae*, *Arabidopsis thaliana* and *Encephalitozoon cuniculi*), we obtained residue-to-residue correspondence of every amino acid in TBP. A structural alignment was obtained using MUSTANG[75]. This was used as the basis for identifying equivalent residues, which was used to compare various residue-based calculations between the different TBP complexes. The different PDB entries were then structurally aligned in pairs for obtaining their corresponding residues and their respective RMSDs were computed using the "bio3d" package in R.

TBP-induced DNA bending. The bending angles of the different DNA sequences in the TBP:DNA complexes were calculated as the angle between the center of atoms of the first and last base pair in the motifs respectively, and the dyad axis of the motif using the "bio3d" package in R.

Promoter DNA shape. The intrinsic minor groove width (MGW) of all TBS sequences in the promoter context were calculated with DNAShape[76], a highly efficient variant of DNA molecular dynamics (MD) simulations that slide over every possible DNA pentamers (44 replicates on average), while reducing the degrees of freedom of base pair to make this approach computationally feasible. Among the various characteristics that this method computes to characterize structural properties of DNA segments, it calculates the minor groove width (MGW). The MGW highlights the distance between the two opposing strands of the DNA phosphate backbones when perceived from the minor groove side. This measure is informative of the intrinsic tendency of a DNA segment to show a widened or contracted minor groove. We applied this approach to a region of +/-15bp around the TBS (including the TBS) of all yeast promoters investigated in the study. As reference we also calculated the per base pair MGW for free (PDB: 1BNA) and TBP bound structures (PDB: 1CWD) of DNA as obtained from Lavery et al.[77], and averaging the MGW output from Curves+ as described in Zhou et al.[76].

Interface properties of structures of TBP-containing complexes. The atomic co-ordinates of TBP in complex with various DNA sequences of TATA-like and TATA-box[3,70-74] and in complex with Taf1p (4B0A)[78], Mot1p (3OC3)[69] and TFIIA (1RM1) were obtained from the PDB. The DNA interaction surface of TBP (concave surface) is almost exclusively conserved between all these sequences. The structures of TBP in complex with Taf1p (TFIID subunit), Mot1p and TFIIA are all from yeast and have very high sequence conservation (see **Supplementary Fig. 4A**). The PDB files were processed to only include TBP and the interaction partner of interest. For all the TBP-DNA complexes and the complexes of TBP with Taf1p and Mot1p (both monomeric proteins) this was straightforward. However the structure of TBP in complex with TFIIA also hosts DNA, which was first removed. The interfaces of TBP with the protein or DNA interaction partners were then "repaired" using FoldX 3.0[79] with standard parameters. This identifies those residues with forbidden torsion angles, van der Waals clashes or total energy and corrects them. This step is especially important to set the structures of the complexes on the same energetic "footing" because different groups solved them using different refinement parameters over the past decades. Furthermore this was important, as removing some of the interaction partners, such as in the case of the TFIIA:TBP complex (1RM1) required readjustments of some side chains. The atomic contacts between TBP and its interactions partners where then characterized with the internal module of the Chimera software[80]. Furthermore, the accessible surface area (ASA) of TBP and the buried surface area (BSA) upon complex formation was calculated using the Hotregions webserver[81], which employs naccess (http://www.bioinf.manchester.ac.uk/naccess/) internally. Finally, for the different complexes containing TBP, the energy contributions of the interfaces were quantitatively estimated using FoldX 3.0[79] using standard parameters.

We proceeded in slightly different manner for the TBP:DNA complexes compared to the TBP-interacting factor complexes. For each of the TBP:DNA complexes the total interface energy was calculated using the "AnalyseComplex" command in the Foldx 3.0. For the energy contributions in the complexes of TBP with other factors (i.e. with Mot1p, Taf1p and TFIIA) we calculated energy contributions on a per residue basis using the "SequenceDetail" function in Foldx 3.0. This was important to (i) distinguish between the energy contributions from the different interfaces of TBP (i.e. the concave vs. the convex interfaces) and (ii) to compare the energy contributions of the subset of TBP residues that have an atomic interaction in common with different factors (i.e. interaction in common between or unique to factors). In our analysis of factor interactions, we wanted to consider the scenario where TBP is already present at the promoter, and for this we did not include TBP residues that interacted with the DNA in our analysis. Hence the convex surface was defined as being composed of those TBP residues that do not interact with the DNA (as determined from one of the TBP:DNA complexes, i.e. 1YTB). The energy contribution to the total energy of complex formation on the convex surface was then calculated as follows: the free

energy of every residue in isolation and in complex with TBP were determined and the sum of the per residue differences in free energy between the two states was calculated. When a residue from Mot1p, TFIIA or Taf1p interacted with both a TBP residue unique to an interaction and involved in interaction that is shared between different factors, the energy contribution was taken into account for both. This then represented the measure of the estimated free energy on complex formation.

Turnover as a measure of dynamics of TBP at the promoter. TBP turnover data at 542 gene promoters were obtained from van Werven et al.[82]. The turnover of TBP at their genomic sites of action was determined by (i) expressing an additional copy of TBP under an inducible promoter with a different tag than that of the wild-type copy under the native promoter and (ii) performing pull down experiments for the respective tags over a time period of 30 min. The authors used a linear regression across the ratio of tags pulled down at the different time points, where the slope represents the TBP turnover. We split the turnover values into two bins (low TBP turnover and high TBP turnover) around at the median. TBP turnover is defined as the rate at which a new molecule of TBP binds at the promoter after an old one has been displaced. The timescale of TBP turnover in this work is on the order of 20 minutes, which cannot capture faster TBP dynamics[83], such as those determined in recent FRAP experiments[84], which were in the seconds timescale.

Testing for statistical significance, correction for multiple testing and visualization of distributions. Statistical analysis was done using the R statistical package. Statistical significance was assessed using the Wilcoxon-sum rank test when comparing distributions and the Chi^2 test when comparing enrichments. Distributions were represented by box plots (outliers not shown for visual clarity). The Mann–Whitney test (or Wilcoxon rank sum test) was used in order to assess whether two samples were from the same population. It is a non-parametric test and does not assume a defined distribution. It consists of first ranking all the observations over the two samples, assigning the smallest value a rank of 1, the next largest a rank of 2, until the largest has the rank equaling the sum of observations. The ranks are then summed up separately for each sample and then their Mann–Whitney statistics are calculated respectively (U and U'). When values are from the same population, U and U' will be similar, yet if either U or U' exceed the critical value for statistical significance, the samples are most likely from different populations. Statistical tests were corrected for multiple testing using the Benjamini & Hochberg method. The Chi^2 test for goodness of fit compares observed ratios to expected ratios for nominal scale data. It helps to assess whether there are significant differences between the expected frequencies and observed frequencies in one or more categories. Boxplots are a visual representation of distributions, highlighting informative statics. The median value for each sample is shown with a horizontal black line. Boxes enclose values between the first and third quartile. The Interquartile range (IQR) is calculated by subtracting the first quartile from the third quartile. All values that are 1.5× IQR lower than the first quartile or 1.5× IQR greater than the third quartile are considered to be outliers and were removed only from the figures to improve visualization.

Markov chain modeling of promoter states and gene expression noise. We performed discrete-time stochastic modeling of gene expression to determine the impact of affinity, competition and residence time of TBP on noise. We used Markov Chains (MC) to model a graph based on our findings with 5 distinct microstates: free promoter (f), TBP:TBS (T), TBS:TBP:Mot1p (M), TBS:TBP:SAGA (S) and TBS:TBP:TFIID (D). The transition probabilities to switch between the microstates were chosen to be reflective of the cellular conditions in yeast cells (see below). Every simulation was conducted for 150 time points and for 500 cells. To account for increased expression (number of transcripts produced per unit time or amplitude) for genes under SAGA regulation [32,45], we modeled an increased rate of expression when a promoter was in the SAGA:TBP microstate compared to the TFIID:TBP microstate at a ratio of 3 to 1 in all simulations. This increase in amplitude would better model the high expression levels of TATA-box genes. The simulations were robust in terms of their noise when varying this parameter. Together with the duration a promoter spends in a given ON state, as modeled by the Markov Chain, the amplitude and duration metrics inform about the total burst size. In this sense, setting the amplitude to 4 for the SAGA complex can be interpreted as TFIID being assembled around 4 times slower in comparison. We did not explicitly model degradation. At the end of the simulation, the total expression levels per cell and the variation thereof in the simulated population of cells was quantified using the coefficient of variation (CV). We used CV to quantify the simulated data as opposed to the distance from the median CV (DM, as used in the rest of the study) as the simulation was done for a single gene and the DM measure is used to normalize the impact of protein abundance across genes. Markov chains were computed using the 'markovchain' R library.

Simulating the impact of TBS affinity for TBP on gene expression noise. The transition probability that describes the situation where a free TBS is bound by TBP ($P_{f->T}$) was a main variable in our analyses. We varied this probability to model the intrinsic affinity of different TBS sequences for TBP (based on the PBM data). Thus, an increasing probability would be reflective of an increasing affinity for the TBP. We varied the $P_{f->T}$ at increments of 0.05 from 0.05 to 0.70 to test this. We used the PBM data to estimate feasible differences in intrinsic affinity of a TATA-like sequence ($P_{f->T} = 0.05$) vs. a TATA-box sequence ($P_{f->T} = 0.4$)[66]. The transition probability for a free TBS to be bound by TFIID was modeled to be constant at $P_{f->D} = 0.25$ irrespective of the intrinsic affinity of the TBS. We made this choice based on the observation that (a) TATA-box containing promoters can be occupied *in vivo* by both TFIID and SAGA[22,85] and (b) TFIID bind to both TATA-box and TATA-like promoters at comparable levels (**Fig. 5d**). Finally, to ensure that the transition probabilities sum up to 1, the probability to remain in the free state was adjusted (i.e. $P_{f->f} = 1- P_{f->T} + P_{f->D}$).

Simulating the impact of competition between Mot1p and SAGA on gene expression noise. To model the competition of Mot1p and SAGA form the TBP:TBS complex, we assigned transition probabilities $P_{T\text{->}M}$ and $P_{T\text{->}S}$ respectively. They were quantified by a metric C_{MS}, which is defined as the ratio of $P_{T\text{->}M}$ over $P_{T\text{->}M} + P_{T\text{->}S}$ and has a value between 0.1 and 0.9 indicating the extreme cases where either SAGA outcompetes Mot1p or Mot1p outcompetes SAGA respectively. To test the impact of this variable on gene expression noise, we varied the C_{MS} in a range of 0.1 to 0.9 at increments of 0.1. The transition probability of $P_{T\text{->}T}$ was modeled as 0.1 as the TBP:TBS complex is generally not long lived in the yeast cells[28], and was kept constant in all simulations.

Simulating the impact of residence time of TFIID, Mot1p and SAGA on gene expression noise. Values for the $P_{D\text{->}D}$, $P_{S\text{->}S}$, and $P_{M\text{->}M}$ transition probabilities, which are indicative of residence times of these complexes at the promoter, were chose to reflect the order of stability that would be expected in yeast cells ($P_{M\text{->}M} < P_{S\text{->}S} < P_{D\text{->}D}$). Thus $P_{M\text{->}M}$ was set to 0.3, which is consistent with its biochemical function to evict TBP from promoters[28]. $P_{S\text{->}S}$ was set to 0.5 as it is a stabilizing co-activator, but leads to less stable complexes than TFIID[86], and $P_{D\text{->}D}$ was set to 0.7, reflective of the low TBP turnover rates that occurs at TFIID regulated genes (which are more likely to have a TATA-like sequence). This value was chosen as until now it remains unknown how TFIID is removed from a promoter once bound. The latter was kept constant in all simulations. For each of these transition probabilities, the complement were adjusted to reach ensure that they sum up to 1 (i.e. $P_{M\text{->}f} = 1 - P_{M\text{->}M}$, $P_{S\text{->}f} = 1 - P_{S\text{->}S}$ and $P_{D\text{->}f} = 1 - P_{D\text{->}D}$).

Deletion of Spt3 and generation of the GFP tagged strains. We deleted the SPT3 subunit of the SAGA complex from the MATα haploid Yeast strain Y6545[87] using nourseothricin (Nat) resistance plasmid pAG35[88]. Synthetic genetic array (SGA) technique was performed between ΔSpt3::Nat^r against the GFP collection (::HIS3; the library was a kind gift from J. Weissman, University of California, San Francisco, San Francisco, CA; Huh et al.[89] Mating was performed on rich media plates, and selection for diploid cells was performed on plates with clonNAT Nourseothricin (Werner) and lacking HIS. Sporulation was then induced by transferring cells to nitrogen starvation plates for 5 days. Haploid cells containing all desired mutations were selected by transferring cells to plates containing all selection markers alongside the toxic amino acid derivatives Canavanine and Thialysine (Sigma-Aldrich) to select against remaining diploids and lacking Leucine to select for only spores with an "a" mating type (Tong et al.[87]; Cohen and Schuldiner[90]). SGA procedure was validated by inspecting representative strains for the presence of the GFP-tagged strains and for the deletion of Spt3 by PCR. To manipulate the collection in high-density format (384), we used a RoToR bench top colony arrayer (Singer Instruments).

Protocol for Flowcytometry. WT and SPT3 deleted GFP-tagged yeast strains (see details below) were measured using flow cytometry. The comparison between the fluorescence emitted by WT GFP-tagged strain (with SPT3) and the knockout shows the impact of the deletion. To process the cytometry data, we followed the protocols from Newman et al[62], Weinberger et al[45] and Hornung et al[60]. Cells were incubated in YPD medium at 30°C overnight to stationary phase, then diluted to an O.D. of 0.01 before growing for another 5–6 hours prior to the measurement. We used a LSRII flow cytometer to measure fluoresence in standard mode at a velocity of 1–1.5 μl/s. GFP was excited at 488 nm and the fluorescence was collected through a 505 nm long-pass filter and 525 nm band-pass filter (Chroma Technology). Thousands of events were recorded from each well in the plate. The flow cytometry experiments were repeated in duplicates. The processing of the raw data was performed as reported before[45]. First it consisted of filtering observations with extreme forward scattering values (0 < SSC-A < 218-1 and 0 < FSC-A < 218-1), and times of data collection. Then we discarded the measurements in the top and bottom 5% in terms of the scattering measured. In order to identify the subpopulation of small cells that did not bud, the measurements were gated to have a total scattering (SSC-A x FSC) below a quintile cutoff value of 0.5. To correct for the effect of size on GFP fluorescence we defined a linear model (GFP ~ FSC-A + SSC-A). The size corrected GFP values were obtained dividing the square of the raw GFP fluorescence measurements by the fitted values of the linear regression. The mean of the residuals of the linear regression indicate the standard deviation of the measurements. The noise or coefficient of variation (C.V.) was calculated with the corrected values dividing the standard deviation by the mean of GFP fluorescence. To estimate the reproducibility of the measurement, the two replicates for the WT, the SPT3 knockout were averaged and the standard error was estimated.

SUPPLEMENTARY REFERENCES

1. Lifton, R.P., Goldberg, M.L., Karp, R.W. & Hogness, D.S. The organization of the histone genes in Drosophila melanogaster: functional and evolutionary implications. *Cold Spring Harb Symp Quant Biol* **42 Pt 2**, 1047-51 (1978).
2. Mishoe, H., Brady, J.N., Lancz, G. & Salzman, N.P. In vitro transcription initiation by purified RNA polymerase II within the adenovirus 2 major late promoter region. *J Biol Chem* **259**, 2236-42 (1984).
3. Patikoglou, G.A. et al. TATA element recognition by the TATA box-binding protein has been conserved throughout evolution. *Genes Dev* **13**, 3217-30 (1999).
4. Basehoar, A.D., Zanton, S.J. & Pugh, B.F. Identification and distinct regulation of yeast TATA box-containing genes. *Cell* **116**, 699-709 (2004).
5. van Werven, F.J. et al. Cooperative action of NC2 and Mot1p to regulate TATA-binding protein function across the genome. *Genes Dev* **22**, 2359-69 (2008).
6. Rhee, H.S. & Pugh, B.F. Genome-wide structure and organization of eukaryotic pre-initiation complexes. *Nature* **483**, 295-301 (2012).
7. Huisinga, K.L. & Pugh, B.F. A genome-wide housekeeping role for TFIID and a highly regulated stress-related role for SAGA in Saccharomyces cerevisiae. *Mol Cell* **13**, 573-85 (2004).
8. Berger, M.F. et al. Compact, universal DNA microarrays to comprehensively determine transcription-factor binding site specificities. *Nat Biotechnol* **24**, 1429-35 (2006).
9. Bonham, A.J., Neumann, T., Tirrell, M. & Reich, N.O. Tracking transcription factor complexes on DNA using total internal reflectance fluorescence protein binding microarrays. *Nucleic Acids Res* **37**, e94 (2009).
10. Raser, J.M. & O'Shea, E.K. Control of stochasticity in eukaryotic gene expression. *Science* **304**, 1811-4 (2004).
11. Tirosh, I. & Barkai, N. Two strategies for gene regulation by promoter nucleosomes. *Genome Res* **18**, 1084-91 (2008).
12. Gietl, A. et al. Eukaryotic and archaeal TBP and TFB/TF(II)B follow different promoter DNA bending pathways. *Nucleic Acids Res* **42**, 6219-31 (2014).
13. Rohs, R., Sklenar, H. & Shakked, Z. Structural and energetic origins of sequence-specific DNA bending: Monte Carlo simulations of papillomavirus E2-DNA binding sites. *Structure* **13**, 1499-509 (2005).
14. Schreiber, G., Haran, G. & Zhou, H.X. Fundamental aspects of protein-protein association kinetics. *Chem Rev* **109**, 839-60 (2009).
15. Kiefhaber, T., Bachmann, A. & Jensen, K.S. Dynamics and mechanisms of coupled protein folding and binding reactions. *Curr Opin Struct Biol* **22**, 21-9 (2012).
16. El Hassan, M.A. & Calladine, C.R. Two distinct modes of protein-induced bending in DNA. *J Mol Biol* **282**, 331-43 (1998).
17. McConnell, K.J. & Beveridge, D.L. Molecular dynamics simulations of B '-DNA: sequence effects on A-tract-induced bending and flexibility. *J Mol Biol* **314**, 23-40 (2001).
18. Pardo, L., Campillo, M., Bosch, D., Pastor, N. & Weinstein, H. Binding mechanisms of TATA box-binding proteins: DNA kinking is stabilized by specific hydrogen bonds. *Biophys J* **78**, 1988-96 (2000).
19. Flatters, D. & Lavery, R. Sequence-dependent dynamics of TATA-Box binding sites. *Biophys J* **75**, 372-81 (1998).
20. Martinez, E. et al. Core promoter-specific function of a mutant transcription factor TFIID defective in TATA-box binding. *Proc Natl Acad Sci U S A* **92**, 11864-8 (1995).
21. Burley, S.K. & Roeder, R.G. Biochemistry and structural biology of transcription factor IID (TFIID). *Annu Rev Biochem* **65**, 769-99 (1996).
22. Cianfrocco, M.A. et al. Human TFIID binds to core promoter DNA in a reorganized structural state. *Cell* **152**, 120-31 (2013).
23. Zentner, G.E. & Henikoff, S. Mot1 redistributes TBP from TATA-containing to TATA-less promoters. *Mol Cell Biol* **33**, 4996-5004 (2013).
24. Holstege, F.C. et al. Dissecting the regulatory circuitry of a eukaryotic genome. *Cell* **95**, 717-28 (1998).
25. Geisberg, J.V. & Struhl, K. Quantitative sequential chromatin immunoprecipitation, a method for analyzing co-occupancy of proteins at genomic regions in vivo. *Nucleic Acids Res* **32**, e151 (2004).
26. Struhl, K. *Interpreting chromatin immunoprecipitation experiments.*, (Cell Press, Cambridge, MA, 2007).
27. Jothi, R. et al. Genomic analysis reveals a tight link between transcription factor dynamics and regulatory network architecture. *Mol Syst Biol* **5**, 294 (2009).
28. Auble, D.T. The dynamic personality of TATA-binding protein. *Trends Biochem Sci* **34**, 49-52 (2009).
29. Arnett, D.R., Jennings, J.L., Tabb, D.L., Link, A.J. & Weil, P.A. A proteomics analysis of yeast Mot1p protein-protein associations: insights into mechanism. *Mol Cell Proteomics* **7**, 2090-106 (2008).
30. Rufiange, A., Jacques, P.E., Bhat, W., Robert, F. & Nourani, A. Genome-wide replication-independent histone H3 exchange occurs predominantly at promoters and implicates H3 K56 acetylation and Asf1. *Mol Cell* **27**, 393-405 (2007).

31. Schluesche, P., Stelzer, G., Piaia, E., Lamb, D.C. & Meisterernst, M. NC2 mobilizes TBP on core promoter TATA boxes. *Nat Struct Mol Biol* **14**, 1196-201 (2007).
32. Zenklusen, D., Larson, D.R. & Singer, R.H. Single-RNA counting reveals alternative modes of gene expression in yeast. *Nat Struct Mol Biol* **15**, 1263-71 (2008).
33. Bhaumik, S.R. Distinct regulatory mechanisms of eukaryotic transcriptional activation by SAGA and TFIID. *Biochim Biophys Acta* **1809**, 97-108 (2011).
34. Moyle-Heyrman, G., Viswanathan, R., Widom, J. & Auble, D.T. Two-step mechanism for modifier of transcription 1 (Mot1) enzyme-catalyzed displacement of TATA-binding protein (TBP) from DNA. *J Biol Chem* **287**, 9002-12 (2012).
35. Dadiani, M. et al. Two DNA-encoded strategies for increasing expression with opposing effects on promoter dynamics and transcriptional noise. *Genome Res* **23**, 966-76 (2013).
36. Segal, E. & Widom, J. From DNA sequence to transcriptional behaviour: a quantitative approach. *Nat Rev Genet* **10**, 443-56 (2009).
37. Sanchez, A., Choubey, S. & Kondev, J. Regulation of noise in gene expression. *Annu Rev Biophys* **42**, 469-91 (2013).
38. Pelechano, V., Wei, W. & Steinmetz, L.M. Extensive transcriptional heterogeneity revealed by isoform profiling. *Nature* **497**, 127-31 (2013).
39. Tirosh, I., Berman, J. & Barkai, N. The pattern and evolution of yeast promoter bendability. *Trends Genet* **23**, 318-21 (2007).
40. Hall, D.B. & Struhl, K. The VP16 activation domain interacts with multiple transcriptional components as determined by protein-protein cross-linking in vivo. *J Biol Chem* **277**, 46043-50 (2002).
41. Papai, G., Weil, P.A. & Schultz, P. New insights into the function of transcription factor TFIID from recent structural studies. *Curr Opin Genet Dev* **21**, 219-24 (2011).
42. Liu, W.L. et al. Structures of three distinct activator-TFIID complexes. *Genes Dev* **23**, 1510-21 (2009).
43. Lehner, B. Conflict between noise and plasticity in yeast. *PLoS Genet* **6**, e1001185 (2010).
44. Zaugg, J.B. & Luscombe, N.M. A genomic model of condition-specific nucleosome behavior explains transcriptional activity in yeast. *Genome Res* **22**, 84-94 (2012).
45. Weinberger, L. et al. Expression noise and acetylation profiles distinguish HDAC functions. *Mol Cell* **47**, 193-202 (2012).
46. Coulon, A., Chow, C.C., Singer, R.H. & Larson, D.R. Eukaryotic transcriptional dynamics: from single molecules to cell populations. *Nat Rev Genet* **14**, 572-84 (2013).
47. Salari, R. et al. Teasing apart translational and transcriptional components of stochastic variations in eukaryotic gene expression. *PLoS Comput Biol* **8**, e1002644 (2012).
48. Maheshri, N. & O'Shea, E.K. Living with noisy genes: how cells function reliably with inherent variability in gene expression. *Annu Rev Biophys Biomol Struct* **36**, 413-34 (2007).
49. Drachkova, I. et al. The mechanism by which TATA-box polymorphisms associated with human hereditary diseases influence interactions with the TATA-binding protein. *Hum Mutat* **35**, 601-8 (2014).
50. Savinkova, L. et al. An experimental verification of the predicted effects of promoter TATA-box polymorphisms associated with human diseases on interactions between the TATA boxes and TATA-binding protein. *PLoS One* **8**, e54626 (2013).
51. Savinkova, L.K. et al. TATA box polymorphisms in human gene promoters and associated hereditary pathologies. *Biochemistry (Mosc)* **74**, 117-29 (2009).
52. Sharma, S.V. et al. A chromatin-mediated reversible drug-tolerant state in cancer cell subpopulations. *Cell* **141**, 69-80 (2010).
53. Cohen, A.A. et al. Dynamic proteomics of individual cancer cells in response to a drug. *Science* **322**, 1511-6 (2008).
54. Gupta, P.B. et al. Stochastic state transitions give rise to phenotypic equilibrium in populations of cancer cells. *Cell* **146**, 633-44 (2011).
55. Dar, R.D., Hosmane, N.N., Arkin, M.R., Siliciano, R.F. & Weinberger, L.S. Screening for noise in gene expression identifies drug synergies. *Science* **344**, 1392-6 (2014).
56. Pijnappel, W.W. et al. A central role for TFIID in the pluripotent transcription circuitry. *Nature* **495**, 516-9 (2013).
57. Blake, W.J. et al. Phenotypic consequences of promoter-mediated transcriptional noise. *Mol Cell* **24**, 853-65 (2006).
58. Murphy, K.F., Adams, R.M., Wang, X., Balazsi, G. & Collins, J.J. Tuning and controlling gene expression noise in synthetic gene networks. *Nucleic Acids Res* **38**, 2712-26 (2010).
59. Mogno, I., Vallania, F., Mitra, R.D. & Cohen, B.A. TATA is a modular component of synthetic promoters. *Genome Res* **20**, 1391-7 (2010).
60. Hornung, G. et al. Noise-mean relationship in mutated promoters. *Genome Res* **22**, 2409-17 (2012).
61. Bar-Even, A. et al. Noise in protein expression scales with natural protein abundance. *Nat Genet* **38**, 636-43 (2006).
62. Newman, J.R. et al. Single-cell proteomic analysis of S. cerevisiae reveals the architecture of biological noise. *Nature* **441**, 840-6 (2006).

63. Swain, P.S., Elowitz, M.B. & Siggia, E.D. Intrinsic and extrinsic contributions to stochasticity in gene expression. *Proc Natl Acad Sci U S A* **99**, 12795-800 (2002).
64. Lopez-Maury, L., Marguerat, S. & Bahler, J. Tuning gene expression to changing environments: from rapid responses to evolutionary adaptation. *Nat Rev Genet* **9**, 583-93 (2008).
65. Mavrich, T.N. et al. A barrier nucleosome model for statistical positioning of nucleosomes throughout the yeast genome. *Genome Res* **18**, 1073-83 (2008).
66. Zhu, C. et al. High-resolution DNA-binding specificity analysis of yeast transcription factors. *Genome Res* **19**, 556-66 (2009).
67. Chasman, D.I., Flaherty, K.M., Sharp, P.A. & Kornberg, R.D. Crystal structure of yeast TATA-binding protein and model for interaction with DNA. *Proc Natl Acad Sci U S A* **90**, 8174-8 (1993).
68. Nikolov, D.B. & Burley, S.K. 2.1 A resolution refined structure of a TATA box-binding protein (TBP). *Nat Struct Biol* **1**, 621-37 (1994).
69. Wollmann, P. et al. Structure and mechanism of the Swi2/Snf2 remodeller Mot1 in complex with its substrate TBP. *Nature* **475**, 403-7 (2011).
70. Juo, Z.S. et al. How proteins recognize the TATA box. *J Mol Biol* **261**, 239-54 (1996).
71. Nikolov, D.B. et al. Crystal structure of a human TATA box-binding protein/TATA element complex. *Proc Natl Acad Sci U S A* **93**, 4862-7 (1996).
72. Kim, J.L., Nikolov, D.B. & Burley, S.K. Co-crystal structure of TBP recognizing the minor groove of a TATA element. *Nature* **365**, 520-7 (1993).
73. Kim, J.L. & Burley, S.K. 1.9 A resolution refined structure of TBP recognizing the minor groove of TATAAAAG. *Nat Struct Biol* **1**, 638-53 (1994).
74. Kim, Y., Geiger, J.H., Hahn, S. & Sigler, P.B. Crystal structure of a yeast TBP/TATA-box complex. *Nature* **365**, 512-20 (1993).
75. Konagurthu, A.S., Whisstock, J.C., Stuckey, P.J. & Lesk, A.M. MUSTANG: a multiple structural alignment algorithm. *Proteins* **64**, 559-74 (2006).
76. Zhou, T. et al. DNAshape: a method for the high-throughput prediction of DNA structural features on a genomic scale. *Nucleic Acids Res* **41**, W56-62 (2013).
77. Lavery, R., Moakher, M., Maddocks, J.H., Petkeviciute, D. & Zakrzewska, K. Conformational analysis of nucleic acids revisited: Curves+. *Nucleic Acids Res* **37**, 5917-29 (2009).
78. Anandapadamanaban, M. et al. High-resolution structure of TBP with TAF1 reveals anchoring patterns in transcriptional regulation. *Nat Struct Mol Biol* **20**, 1008-14 (2013).
79. Schymkowitz, J. et al. The FoldX web server: an online force field. *Nucleic Acids Res* **33**, W382-8 (2005).
80. Pettersen, E.F. et al. UCSF Chimera--a visualization system for exploratory research and analysis. *J Comput Chem* **25**, 1605-12 (2004).
81. Cukuroglu, E., Gursoy, A. & Keskin, O. HotRegion: a database of predicted hot spot clusters. *Nucleic Acids Res* **40**, D829-33 (2012).
82. van Werven, F.J., van Teeffelen, H.A., Holstege, F.C. & Timmers, H.T. Distinct promoter dynamics of the basal transcription factor TBP across the yeast genome. *Nat Struct Mol Biol* **16**, 1043-8 (2009).
83. Poorey, K. et al. Measuring chromatin interaction dynamics on the second time scale at single-copy genes. *Science* **342**, 369-72 (2013).
84. Sprouse, R.O. et al. Regulation of TATA-binding protein dynamics in living yeast cells. *Proc Natl Acad Sci U S A* **105**, 13304-8 (2008).
85. Juven-Gershon, T., Cheng, S. & Kadonaga, J.T. Rational design of a super core promoter that enhances gene expression. *Nat Methods* **3**, 917-22 (2006).
86. Han, Y., Luo, J., Ranish, J. & Hahn, S. Architecture of the Saccharomyces cerevisiae SAGA transcription coactivator complex. *EMBO J* **33**, 2534-46 (2014).
87. Tong, A.H. et al. Systematic genetic analysis with ordered arrays of yeast deletion mutants. *Science* **294**, 2364-8 (2001).
88. Goldstein, A.L. & McCusker, J.H. Three new dominant drug resistance cassettes for gene disruption in Saccharomyces cerevisiae. *Yeast* **15**, 1541-53 (1999).
89. Huh, W.K. et al. Global analysis of protein localization in budding yeast. *Nature* **425**, 686-91 (2003).
90. Cohen, Y. & Schuldiner, M. Advanced methods for high-throughput microscopy screening of genetically modified yeast libraries. *Methods Mol Biol* **781**, 127-59 (2011).
